# Desmoglein-2 harnesses a PDZ-GEF2/Rap1 signaling axis to control cell spreading and focal adhesions independent of cell–cell adhesion

**DOI:** 10.1038/s41598-021-92675-1

**Published:** 2021-06-24

**Authors:** W. Tucker Shelton, S. Madison Thomas, Hunter R. Alexander, C. Evan Thomes, Daniel E. Conway, Adi D. Dubash

**Affiliations:** 1 Department of Biology, Furman University, 3300 Poinsett Highway, Greenville, SC 29613 USA; 2 Department of Biomedical Engineering, Virginia Commonwealth University, 601 West Main Street, Richmond, VA 23284 USA

**Keywords:** Cadherins, Desmosomes, Extracellular matrix, Focal adhesion

## Abstract

Desmosomes have a central role in mediating extracellular adhesion between cells, but they also coordinate other biological processes such as proliferation, differentiation, apoptosis and migration. In particular, several lines of evidence have implicated desmosomal proteins in regulating the actin cytoskeleton and attachment to the extracellular matrix, indicating signaling crosstalk between cell–cell junctions and cell–matrix adhesions. In our study, we found that cells lacking the desmosomal cadherin Desmoglein-2 (Dsg2) displayed a significant increase in spreading area on both fibronectin and collagen, compared to control A431 cells. Intriguingly, this effect was observed in single spreading cells, indicating that Dsg2 can exert its effects on cell spreading independent of cell–cell adhesion. We hypothesized that Dsg2 may mediate cell–matrix adhesion via control of Rap1 GTPase, which is well known as a central regulator of cell spreading dynamics. We show that Rap1 activity is elevated in Dsg2 knockout cells, and that Dsg2 harnesses Rap1 and downstream TGFβ signaling to influence both cell spreading and focal adhesion protein phosphorylation. Further analysis implicated the Rap GEF PDZ-GEF2 in mediating Dsg2-dependent cell spreading. These data have identified a novel role for Dsg2 in controlling cell spreading, providing insight into the mechanisms via which cadherins exert non-canonical junction-independent effects.

## Introduction

The desmosome is a transmembrane complex of junctional proteins crucial for maintaining cell–cell adhesion, tissue structure/integrity and resistance to mechanical stress^1–3^. Desmosomes have a tripartite organizational structure composed of three major classes of proteins. The cadherins Desmoglein (Dsg) and Desmocollin (Dsc) connect cells together in the extracellular space, mediated via homophilic and heterophilic interactions of their extracellular domains^4^. On the intracellular face, armadillo proteins Plakoglobin (PG) and Plakophilin (PKP) bind to the cytoplasmic tails of cadherins and form a stable plaque which provides structural support to the desmosome complex^1,5^. Lastly, the plakin protein Desmoplakin (DP) connects the cytoplasmic plaque to the intermediate filament (IF) network, thus providing anchor points for IF fibers and mediating their important role in shock absorption^6^. The adhesive function of desmosomes is particularly important in tissues like the myocardium and epidermis that are exposed to significant and frequent levels of mechanical stress^7^. Considering this critical role for desmosomes in mediating tissue integrity, it is not surprising that mutations in desmosomal cadherins (and other desmosome components) have been linked to a variety of different epidermal disorders (related to skin fragility/blistering, loss of hair, etc.) and also arrhythmogenic cardiomyopathy, which is characterized by loss of cell–cell adhesion and gap junction defects in cardiomyocytes^8,9^.

Beyond this fundamental role in cell–cell adhesion, the past few decades of research have re-characterized desmosomes as junctional signaling centers involved in a wide range of biological processes from differentiation and morphogenesis to cell shape and migration^10,11^. Intriguingly, the non-canonical functions of desmosomal proteins can be mediated by non-junctional effects of these proteins^7,12–15^. We have recently shown that the cytolinker DP harnesses p38 MAPK and Rac1 signaling to control actin cytoskeletal architecture and cell migration, and that these effects of DP are seen in single spreading cells which do not form cell–cell contacts^15^. As desmosomal cadherins are central players in mediating cell–cell attachment, we investigated whether loss of Desmoglein-2 (Dsg2) in A431 epidermal cells would have an effect on the attachment and spreading of single cells on extracellular matrix (ECM). Intriguingly, we observed a significant increase in spreading area for individual Dsg2 knockout cells (Dsg2KO) compared to control A431 cells (A431CT), suggesting that even when Dsg2 is not part of a stable cell–cell junction (i.e., in single spreading cells), it can still exerts signaling effects involved in crosstalk with cell–matrix adhesions. Moreover, we found that loss of Dsg2 results in enhanced phosphorylation of the focal adhesion proteins Paxillin and Focal Adhesion Kinase (FAK), which is indicative of enhanced integrin-mediated engagement to the ECM^16^. Both of these effects are recapitulated during single cell spreading of Dsg2 knockdown HaCaT keratinocytes, confirming that these results are not due to cell type-specific effects of A431 cells or off target effects of CRISPR-mediated knockout. Interestingly, differences in focal adhesion protein phosphorylation were lost in confluent monolayers of A431CT and Dsg2KO cells, indicating that the prominent effects of Dsg2 on cell spreading may be masked by other junctional proteins when widespread and uniform cell–cell adhesion is present.

The GTPase Rap1 is a well-established regulator of cell attachment and spreading. Integrin-mediated ECM adhesion can activate Rap1, which leads to a positive feedback loop of inside-out signaling which enhances integrin attachment and formation of focal adhesions^17,18^. We therefore hypothesized that Rap1 may mediate the effects of Dsg2 on cell spreading and focal adhesions. We show that Rap1 activity is elevated in Dsg2KO cells, and that knockdown of Rap1 rescues both the enhanced spreading and elevated focal adhesion protein phosphorylation observed in Dsg2KO cells, confirming the involvement of Rap1 in these Dsg2-mediated effects.

Like most other small GTPases, Rap1 activity is regulated via cyclical switching between an active GTP-bound form and an inactive GDP-bound form^19,20^. Guanine nucleotide exchange factors (GEFs) for Rap enhance its activity by promoting exchange of GDP for GTP, and GTPase-activating proteins (GAPs) trigger Rap’s intrinsic ability to hydrolyze GTP, which decreases its activity. Several Rap GEFs and GAPs have been implicated in modulating ECM attachment and focal adhesion structure^19,20^. Investigating the putative involvement of several major Rap GEFs in Dsg2-mediated cell spreading revealed a significant rescue when PDZ-GEF2 was knocked down, implicating an important role for this GEF in controlling cell spreading via Dsg2.

To further delineate the mechanisms involved in Dsg2-mediated cell spreading, we analyzed two signaling pathways connected to Dsg2 and Rap1 which are known to be involved in cell spreading. We show here that while both Erk and Transforming growth factor β (TGFβ) signaling is elevated in Dsg2KO cells, only inhibition of TGFβ signaling was sufficient to rescue the enhanced spreading and focal adhesion protein phosphorylation in Dsg2KO cells. Furthermore, TGFB2 mRNA (but not TGFB1 or TGFB3 mRNA) and secreted TGFβ2 protein is elevated in Dsg2KO cells, and treatment of A431CT cells with TGFβ2 alone is sufficient to enhance cell spreading and paxillin phosphorylation. Taken together, these data have identified a novel Dsg2-Rap1-TGFβ signaling axis that controls cell spreading. Importantly, these effects of Dsg2 are independent of its central role in mediating desmosomal cell–cell adhesion. These data therefore shed light on non-junctional effects of desmosomal cadherins and highlight the importance of studying crosstalk signaling mechanisms between junctional proteins and cell–matrix adhesions.

## Results

### Loss of Desmoglein-2 enhances spreading of cells on extracellular matrix, independent of cell–cell attachment

Several reports have characterized roles for Desmoglein-2 (Dsg2) in coordinating a wide variety of cellular processes including proliferation, apoptosis, differentiation and cell migration^4,11^. Like other members of the desmosome (such as DP and PKP2), desmosomal cadherins have been shown to control actin cytoskeletal rearrangements^21^, which are necessary for the dynamic cell shape changes involved in processes such as cell–matrix adhesion, spreading and migration^22^. Based on these reports, we hypothesized that loss of Dsg2 would alter spreading of cells on the extracellular matrix (ECM). In particular, our goal was to investigate whether Dsg2 could exert effects on actin cytoskeletal dynamics in single spreading cells, independent of cell–cell contact.

**Figure 1:**
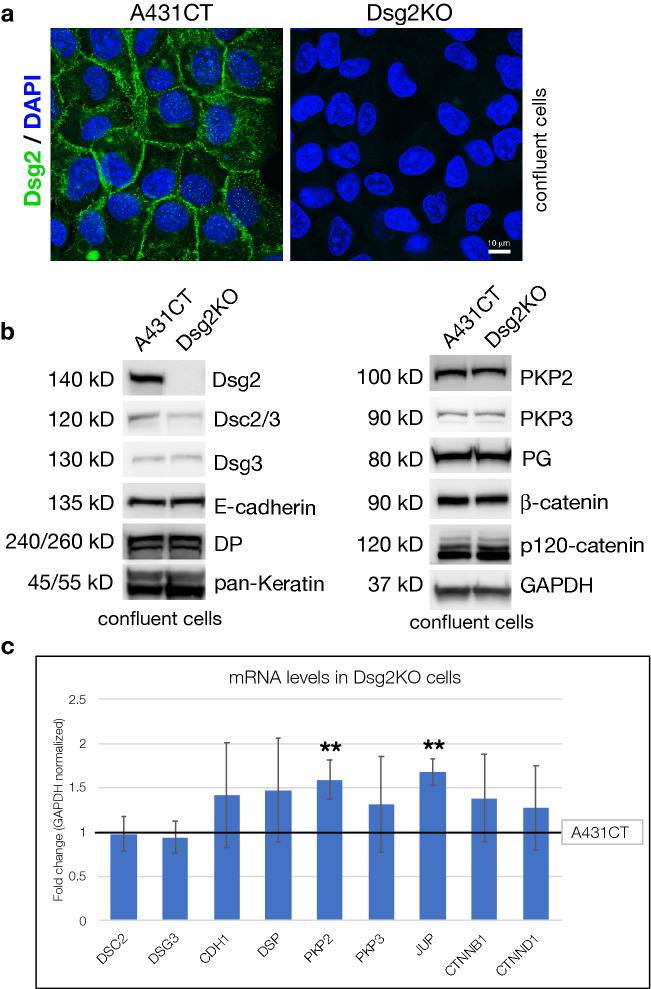
Loss of Desmoglein-2 (Dsg2) does not dramatically perturb expression of other cell–cell junctional proteins in confluent monolayers of cells. (**a**) Control A431 cells (A431CT) and Desmoglein-2 knockout cells (Dsg2KO) growing on coverslips at confluency were fixed and stained for Desmoglein-2 (Dsg2) and nuclei (DAPI). (**b**) A431CT & Dsg2KO cells growing in tissue culture dishes at confluency were processed for SDS-PAGE and blotted for the following junctional proteins: Desmoglein-2 (Dsg2), Desmocollin-2/3 (Dsc2/3), Desmoglein-3 (DG3), E-cadherin, Desmoplakin (DP), pan-Keratin, Plakophilin-2 (PKP2), Plakophilin-3 (PKP3), Plakoglobin (PG), β-catenin, p120-catenin and GAPDH (loading control). Unprocessed blots from this figure are shown in Supplementary Fig. S3. (**c**) Total RNA was isolated from A431CT & Dsg2KO cells, followed by qPCR to analyze mRNA levels of the following genes: Desmocollin-2 (*DSC2*), Desmoglein-3 (*DSG3*), E-cadherin (*CDH1*), Desmoplakin (*DSP*), Plakophilin-2 (*PKP2*), Plakophilin-3 (*PKP3*), Plakoglobin (*JUP*), β-catenin (*CTNNB1*) and p120 catenin (*CTNND1*). Graph represents fold change values of mRNA levels in Dsg2KO cells compared to A431CT cells (reference line), with error bars indicating s.d. **p < 0.01 vs. A431CT.

To investigate these goals, we employed the use of control A431 epidermal cells (A431CT) and Dsg2 knockout A431 cells (Dsg2KO)^23^. Before performing functional analyses, loss of Dsg2 protein expression in Dsg2KO cells was confirmed via immunofluorescence and western blot (Fig. 1a,b). We also analyzed whether loss of Dsg2 affected expression of other cell–cell adhesion proteins found in desmosomes and adherens junctions. In confluent monolayers of cells, Dsg2 knockout did not alter the mRNA levels of other cadherins such as Desmocollin-2 (*DSC2*), Desmoglein-3 (*DSG3*) or E-cadherin (*CDH1*) (Fig. 1c). While the protein levels of Desmocollin-2/3 (Dsc2/3) were partially reduced in Dsg2KO cells, the protein levels of Desmoglein-3 (Dsg3) and E-cadherin were not affected (Fig. 1b). In addition, mRNA and protein levels of other junctional proteins (DP, PKP3, Keratin, β-catenin or p120-catenin) were unperturbed (Fig. 1b,c). While minor ~ 0.5-fold increases in mRNA levels were observed for Plakophilin-2 (PKP2) and Plakoglobin (JUP), no significant change in protein levels was observed for these two desmosomal armadillo proteins (Fig. 1b,c). These data confirm that knockout of Dsg2 does not cause a broad-scale change in expression levels of other cell–cell junction proteins in cells growing in confluent monolayers.

**Figure 2:**
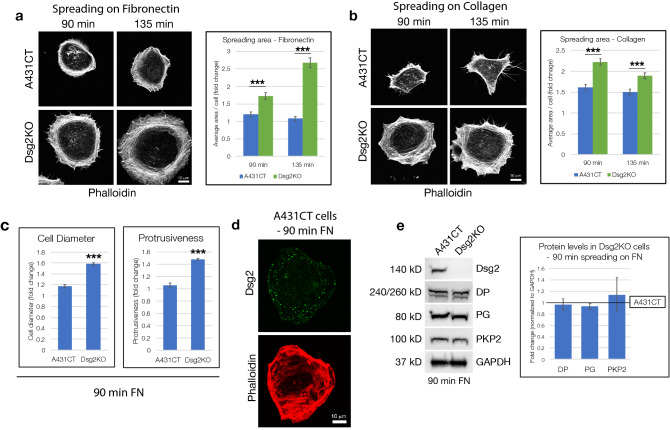
Loss of Desmoglein-2 enhances spreading of single cells on ECM, independent of cell–cell attachment. (**a**,**b**) A431CT and Dsg2KO cells were trypsinized, held in suspension for 30 min and plated at a very low density on to coverslips pre-coated with either fibronectin (*FN*) (**a**) or type I collagen (**b**). Following cell spreading for 90 or 135 min, coverslips were fixed and stained with Phalloidin to visualize F-actin. (**a**,**b**) Graphs represent fold change differences in average area/cell in Dsg2KO cells compared to A431CT cells, with error bars indicating s.e.m. ***p < 0.001 vs. control. (**c**) A431CT and Dsg2KO cells spreading on FN for 90 min were fixed, stained with Phalloidin and DAPI (to visualize F-actin and nuclei) and analyzed for cell diameter and protrusiveness, as described in materials and methods. Error bars represent s.e.m. ***p < 0.001 vs. control. (**d**) A431CT cells spreading on FN for 90 min were fixed and stained for Dsg2 and Actin. (**e**) A431CT and Dsg2KO cells spreading on FN for 90 min were processed for SDS-PAGE and blotted for the following desmosomal proteins: Dsg2, DP, PKP2, PG and GAPDH (loading control). Graph represents fold change differences in protein levels in Dsg2KO cells compared to A431CT cells (reference line), with error bars indicating s.d. (no significant differences were found). Unprocessed blots from this figure are shown in Supplementary Fig. S4.

To analyze the effect of Dsg2 on cell spreading, cultured A431CT and Dsg2KO cells were trypsinized into a single cell solution, held in suspension for 30 min at 37 °C, and plated onto coverslips pre-coated with fibronectin (FN) at a low density to allow for spreading of individual cells, independent of cell–cell contact. Single cells were allowed to spread on FN for 90 and 135 min, fixed and stained with Phalloidin (to visualize F-actin), followed by measurement of cell area using ImageJ. Compared to A431CT cells, Dsg2KO cells demonstrated a statistically significant increase in cell spreading on FN at both 90 and 135 min (Fig. 2a). To confirm that our quantified changes in cell spreading were not simply an artifact of alterations in F-actin staining, we also quantified cell area after treating cells with CellTracker Green CMFDA, a fluorescent dye which transforms into a cell-impermeant fluorescent product once inside cells (Supplementary Fig. S1). These results recapitulated our findings that loss of Dsg2 significantly enhances cell spreading on FN.

To determine whether enhanced spreading of Dsg2KO cells was a FN-specific effect, we next plated cells on collagen-coated coverslips. The increase in spreading area for Dsg2KO cells was replicated on collagen at both 90 and 135 min (Fig. 2b), suggesting that the ability of Dsg2 to regulate cell spreading was not due to outside-in signaling unique to FN attachment or FN-specific integrins. In addition to cell area, cell diameter and protrusiveness (measured as distance from nucleus to the edge of lamellipodial protrusions) are also significantly higher in actively spreading Dsg2KO cells compared to A431CT cells (Fig. 2c). Taken together, these results in single cell spreading assays demonstrate that Dsg2 plays a role in cell spreading and ECM attachment independent of cell–cell attachment. Unlike confluent cells (where Dsg2 is anchored at cell–cell junctions), Dsg2 localization in individually spreading cells occurs in a punctate distribution in the cytoplasm and around the cell periphery (Fig. 2d). Such a distribution for Dsg2 in the absence of stable cell–cell junctions is an expected result, and conforms to what has been described previously for vesicle-mediated transport of Dsg2 to the cell membrane^24,25^. Further analysis of other major desmosomal components (DP, PG, and PKP2) in singly spreading cells demonstrated no significant differences in total protein levels (Fig. 2e), and no dramatic differences in localization (Supplementary Fig. S1).

### Loss of Dsg2 increases phosphorylation of focal adhesion proteins in actively spreading cells

**Figure 3:**
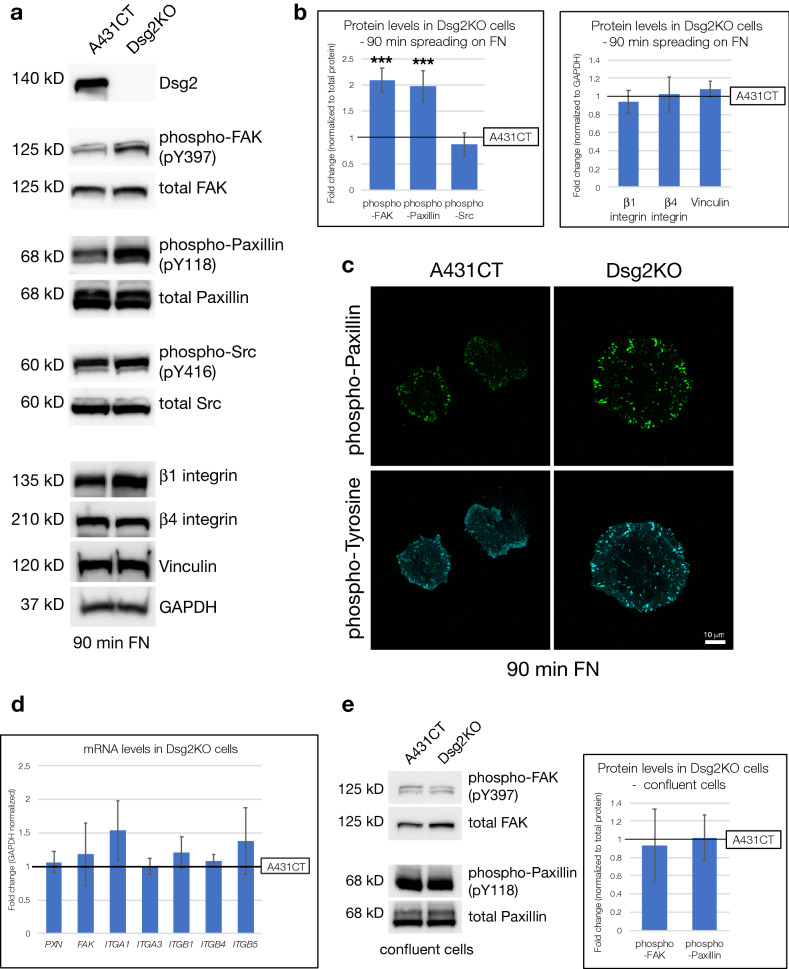
Loss of Dsg2 increases phosphorylation of focal adhesion proteins in singly spreading cells on FN, but not confluent cells. (**a**) A431CT and Dsg2KO cells spreading on FN for 90 min were processed for SDS-PAGE and blotted with the following antibodies: Dsg2, phospho-FAK (pY397) and total FAK, phospho-Paxillin (pY118) and total Paxillin, phospho-Src (pY416) and total Src, β1 and β4 integrin, Vinculin and GAPDH (loading control). **(b)** Densitometric analysis was performed as described in materials and methods. Graphs represent fold change differences in protein levels in Dsg2KO cells compared to A431CT cells (reference line), with error bars indicating s.d. ***p < 0.001 vs. control. (**c**) A431CT and Dsg2KO cells spreading on FN-coated coverslips for 90 min were fixed and stained with phospho-Y118 Paxillin and phospho-Tyrosine antibodies. (**d**) A431CT & Dsg2KO cells were subjected to quantitative PCR to analyze mRNA levels of the following genes: Paxillin (PXN), Focal Adhesion Kinase (FAK), α1 integrin (ITGA1), α3 integrin (ITGA3), β1 integrin (ITGB1), β4 integrin (ITGB4) and β5 integrin (ITGB5). Graph represents fold change values of mRNA levels in Dsg2KO cells compared to A431CT cells (reference line), with error bars indicating s.d. No significant differences were found. (**e**) A431CT and Dsg2KO cells growing in confluent monolayers were processed for SDS-PAGE and blotted with the following antibodies: phospho-FAK (pY397), total FAK, phospho-Paxillin (pY118) and total Paxillin. Graph represents fold change differences in protein levels in Dsg2KO cells compared to A431CT cells (reference line), with error bars indicating s.d. No significant differences were found. Unprocessed blots from this figure are shown in Supplementary Fig. S5.

Considering the effect of Dsg2 on cell spreading dynamics, we next sought to determine whether focal adhesions were altered in Dsg2KO cells. Compared to A431CT cells, Dsg2KO cells in the process of spreading (90 min on FN) demonstrated a significant increase in phosphorylation of Paxillin and FAK, two focal adhesion proteins which are central players in integrin-mediated cell attachment and spreading on ECM (Fig. 3a,b). In contrast to FAK and Paxillin, phosphorylation of Src and expression of Vinculin was not perturbed by loss of Dsg2 (Fig. 3a,b). In addition to these data obtained via western blot, increased intensity of phospho-Paxillin and phospho-tyrosine staining in Dsg2KO cells were also observed via immunofluorescence, confirming elevated phosphorylation of focal adhesion proteins in actively spreading Dsg2KO cells compared to A431CT (Fig. 3c). As Paxillin and FAK are well-known mediators of integrin-dependent ECM adhesion, we analyzed the expression levels of different α and β integrins, a subset of which are shown here. Several prior studies have documented changes in integrin gene expression upon loss of other desmosomal proteins^14,26^. Nevertheless, loss of Dsg2 did not induce changes in protein levels of β1 and β4 integrins during cell spreading (Fig. 3a,b). We also did not observe any changes in mRNA levels of FAK, PXN, or many different integrins tested (ITGA1, ITGA3, ITGB1, ITGB4 and ITGB5), indicating that Dsg2 does not mediate its effect via changes in expression of integrins or other focal adhesion proteins (Fig. 3d).

Analysis of sub-confluent cells growing in culture 24 h after plating on FN demonstrated that Dsg2KO cells were larger and rounder than A431CT cells, as seen during active cell spreading (Supplementary Fig. S2). While focal adhesion structure and distribution (as visualized by staining for total Paxillin) was not dramatically different between sub-confluent cultures of A431CT and Dsg2KO cells, levels of phosphorylated Paxillin were enhanced in Dsg2KO cells, as seen for actively spreading cells (Supplementary Fig. S2). Importantly, in contrast to actively spreading single cells and sub-confluent cultures growing on FN, analysis of confluent monolayers revealed no significant differences in FAK or Paxillin phosphorylation between A431CT and Dsg2KO cells (Fig. 3e). These data indicate that while Dsg2 controls spreading and ECM attachment in singly spreading cells and sub-confluent cultures, this effect is muted in confluent monolayers of cells where robust cell–cell attachment via other junctional proteins may mask the effects of Dsg2.

**Figure 4:**
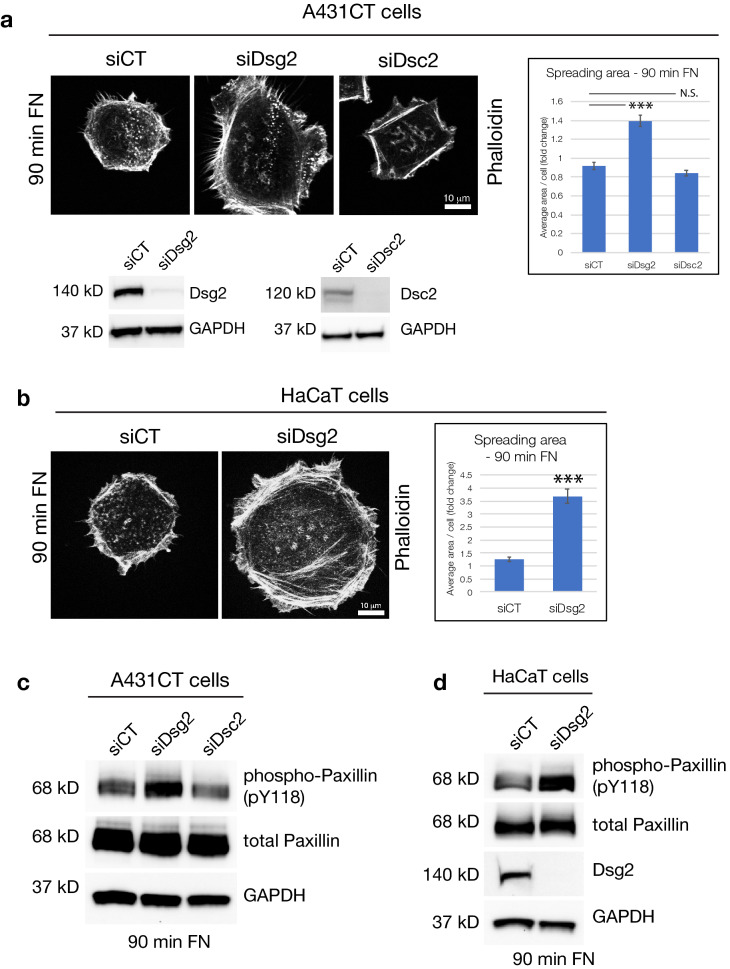
Knockdown of Dsg2, but not Dsc2, increases cell spreading and Paxillin phosphorylation. (**a**) A431 cells were transfected with control siRNA (siCT) or siRNA specific for either Desmoglein-2 (siDsg2) or Desmocollin-2 (siDsc2). 72 h post transfection, cells were either subjected to cell spreading assays on FN (90 min FN) or processed for SDS-PAGE and blotted for Dsg2 or Dsc2 and GAPDH (loading control). Graph represents fold change differences in average area/cell in siDsg2 and siDsc2 cells compared to siCT cells, with error bars indicating s.e.m. ***p < 0.001 vs. control. N.S. = not significant vs. control. (**b**) HaCaT cells were transfected with siCT or siDsg2. 72 h post transfection, cells were subjected to cell spreading assays on FN-coated coverslips (90 min FN). Graph represents fold change differences in average area/cell in siDsg2 cells compared to siCT cells, with error bars indicating s.e.m. ***p < 0.001 vs. control. (**c**,**d**) A431CT cells transfected with siCT, siDsg2 and siDsc2, or HaCaT cells transfected with siCT and siDsg2 were subjected to spreading assays on FN for 90 min and processed for SDS-PAGE and blotted with the following antibodies: phospho-Paxillin (pY118), total Paxillin, Dsg2 and GAPDH. All unprocessed blots from this figure are shown in Supplementary Fig. S6.

To confirm that these findings were not due to off-target effects of CRISPR-mediated knockout, we performed siRNA-mediated knockdown of Dsg2 (siDsg2) in A431CT cells. Like Dsg2KO cells, siDsg2 cells displayed a similar increase in cell spreading area on FN, providing further validation of these results (Fig. 4a). Interestingly, knockdown of Desmocollin-2 (siDsc2) did not produce a similar increase in cell spreading, indicating that this effect is specific to the desmosomal cadherin Dsg2 (Fig. 4a). Increased phosphorylation of Paxillin was also observed in Dsg2 knockdown cells, but not Dsc2 knockdown cells (Fig. 4c). We also sought to evaluate whether these effects are specific to A431 epidermal cancer cells, or whether they also occur in normal, non-transformed keratinocytes. siRNA-mediated knockdown of Dsg2 in HaCaT keratinocytes induces both an increase in cell spreading and paxillin phosphorylation, indicating that these effects of Dsg2 are not cell type specific (Fig. 4b,d).

### Dsg2 effects on cell spreading and focal adhesion protein phosphorylation are mediated by Rap1

**Figure 5:**
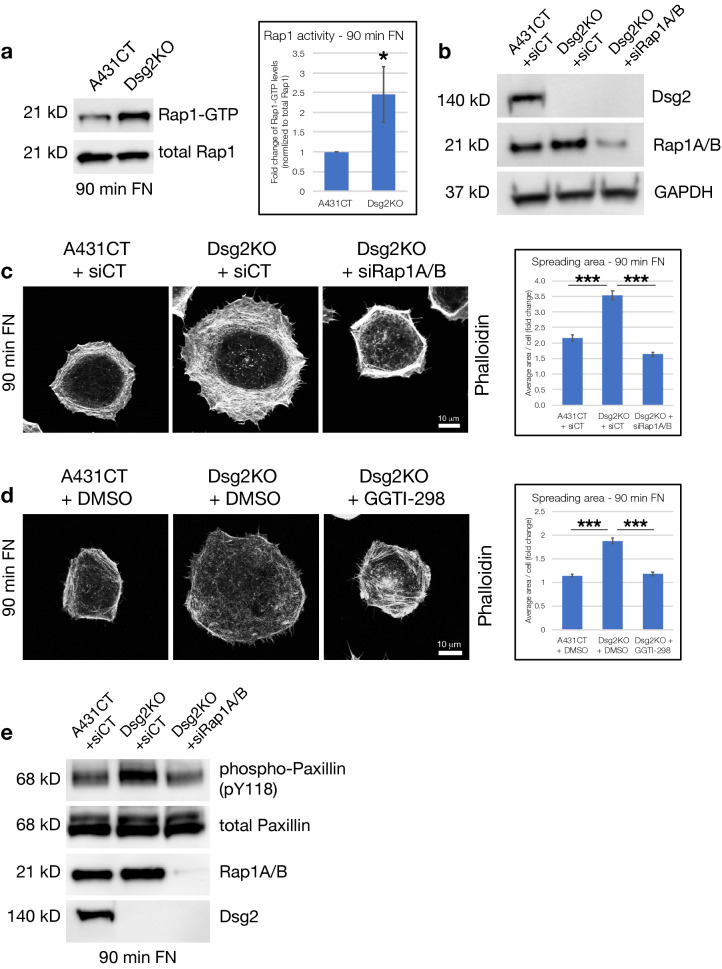
Dsg2 effects on cell spreading and focal adhesion protein phosphorylation are mediated by Rap1. (**a**) A431CT and Dsg2KO cells spreading on *FN* for 90 min were lysed and GST-Ral GDS Rap Binding Domain (RBD) pulldowns performed to precipitate active GTP-bound Rap1 from cellular lysates. Pulldown and total lysate samples were subjected to SDS-PAGE and blotted for Rap1. Graph represents fold change in Rap1 activity (normalized to total Rap1 levels) from three independent experiments, with error bars indicating s.d. *p < 0.05. (**b**) To knockdown Rap1, cells were transfected with Rap1A/B-specific siRNA (siRap1A/B) or non-targeting siRNA being used as a control (siCT). 72 h following transfection, western blots for Dsg2, Rap1A/B and GAPDH (loading control) confirmed efficient knockdown of Rap1A/B. (**c**) A431CT + siCT, Dsg2KO + siCT and Dsg2KO + siRap1A/B cells were subjected to cell spreading on FN for 90 min, fixed and stained with Phalloidin (to visualize F-actin) and spreading area quantified (**d**) A431CT + DMSO (vehicle), Dsg2KO + DMSO and Dsg2KO + GGTI-298 cells (pre-treated for 30 min in suspension) were subjected to cell spreading on FN for 90 min, fixed and stained with Phalloidin (to visualize F-actin) and spreading area quantified (**c**,**d**) Graphs represents average area/cell, with error bars indicating s.e.m. ***p < 0.001. (**e**) A431CT + siCT, Dsg2KO + siCT and Dsg2KO + siRap1A/B cells were subjected to cell spreading on FN for 90 min, processed for SDS-PAGE and blotted with the following antibodies: phospho-Paxillin (pY118), total Paxillin, Rap1A/B and Dsg2. All unprocessed blots from this figure are shown in Supplementary Fig. S7.

To investigate the signaling mechanisms via which Dsg2 controls cell spreading, we first analyzed activity levels of Rap1 GTPase, as it is well known to control both inside-out and outside-in signaling related to cell spreading^17,18^. While loss of Dsg2 did not affect Rap1 expression, analysis of GTP-bound levels of Rap1 by pulldown assays demonstrated a significant increase in Rap1 activity in Dsg2KO cells spreading on *FN* (90 min), compared to control A431CT cells (Fig. 5a). To determine whether altered Rap1 activity may be responsible for the enhanced spreading of Dsg2KO cells, we analyzed spreading dynamics in response to knockdown of Rap1A/B (siRap1A/B). Loss of Rap1A/B expression in Dsg2KO cells (confirmed by western blot in Fig. 5b) caused a significant rescue of cell spreading, compared to Dsg2KO cells alone (Fig. 5c). Inhibition of Rap1 activity in Dsg2KO cells (via the geranylgeranyltransferase inhibitor GGTI-298) also recapitulated these results (Fig. 5d). Moreover, Rap1A/B knockdown was sufficient to rescue the increases in phosphorylation of Paxillin observed in Dsg2KO cells spreading for 90 min on FN (Fig. 5e). These data collectively demonstrate that Dsg2 mediates its effects on cell spreading and focal adhesion protein phosphorylation via Rap1 signaling.

### PDZ-GEF2 mediates Dsg2 effects on cell spreading and focal adhesion protein phosphorylation

**Figure 6:**
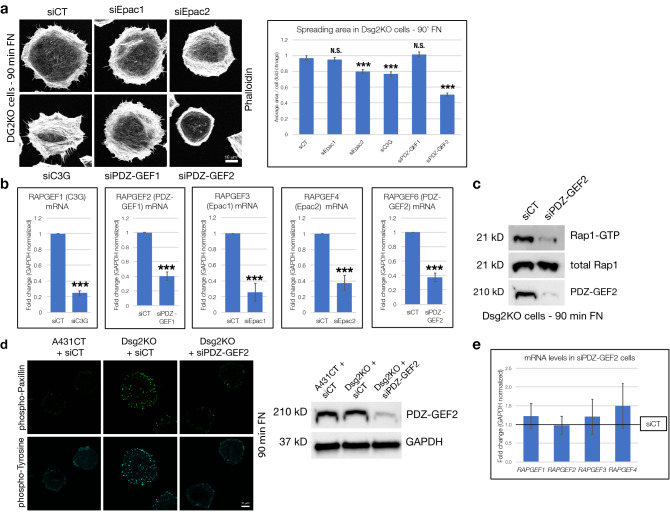
PDZ-GEF2 mediates Dsg2 effects on cell spreading and focal adhesion protein phosphorylation. (**a**) Dsg2KO cells were transfected with either control siRNA (siCT) or gene-specific siRNA for Epac1, Epac2, C3G, PDZ-GEF1 or PDZ-GEF2. 72 h following transfection, samples were subjected to cell spreading (90 min on FN), fixed and stained with Phalloidin (to visualize F-actin) and spreading area quantified. Graph represents average area/cell, with error bars indicating s.e.m. ***p < 0.001 vs. control; N.S. = not significant vs. control. (**b**) Dsg2KO cells were transfected with either control siRNA (siCT) or gene-specific siRNA for RAPGEF1 (C3G), RAPGEF2 (PDZ-GEF1), RAPGEF3 (Epac1), RAPGEF4 (Epac2) or RAPGEF6 (PDZ-GEF2), followed by quantitative PCR to analyze mRNA levels of these Rap GEFs. Graphs represent fold change values of mRNA levels in knockdown cells compared to controls, with error bars indicating s.d. ***p < 0.001 vs. control. (**c**) Dsg2KO cells treated with control siRNA (siCT) or siRNA specific for PDZ-GEF2 (siPDZ-GEF2) were subjected to spreading on FN for 90 min, followed by lysis and pulldowns with GST-Ral GDS Rap Binding Domain (RBD) to precipitate active GTP-bound Rap1 from cellular lysates. Pulldown and total lysate samples were subjected to SDS-PAGE and blotted for Rap1 and PDZ-GEF2*.* (**d**) A431CT + siCT, Dsg2KO + siCT and Dsg2KO + siPDZ-GEF2 cells were subjected to spreading assays and stained with phospho-Paxillin (pY118) and phospho-Tyrosine antibodies. Western blots for PDZ-GEF2 and GAPDH (loading control) confirm efficient knockdown of PDZ-GEF2 in these samples. (**e**) Dsg2KO cells treated with control siRNA (siCT) or siRNA specific for PDZ-GEF2 (siPDZ-GEF2) were subjected to quantitative PCR to analyze mRNA levels of RAPGEF1 (C3G), RAPGEF2 (PDZ-GEF1), RAPGEF3 (Epac1), and RAPGEF4 (Epac2). Graph represents fold change values of mRNA levels in siPDZ-GEF2 knockdown cells compared to siCT cells (reference line), with error bars indicating s.d. No significant differences were found. All unprocessed blots from this figure are shown in Supplementary Fig. S7.

To analyze how Dsg2 controls Rap1 signaling, we examined possible roles for known Rap1 guanine nucleotide exchange factors (GEFs) via a siRNA-mediated knockdown approach. Out of the known Rap GEFs we tested (Epac1, Epac2, C3G, PDZ-GEF1 and PDZ-GEF2), minor changes in spreading were observed upon loss of Epac2 and C3G, but only knockdown of PDZ-GEF2 produced a pronounced (~ 50%) reduction in spreading area of Dsg2KO cells plated on FN for 90 min (Fig. 6a). Efficient knockdown of mRNA levels of each Rap GEF was determined via quantitative PCR (Fig. 6b). As expected from previous reports^27^, knockdown of PDZ-GEF2 in Dsg2KO cells resulted in a reduction of Rap1-GTP levels (Fig. 6c). PDZ-GEF2 knockdown in Dsg2KO cells was sufficient to rescue the increases in phosphorylation of focal adhesion proteins, as visualized by phospho-Paxillin and phospho-Tyrosine staining in cells actively spreading on FN for 90 min (Fig. 6d). In addition, we confirmed that knockdown of PDZ-GEF2 is specific and does not affect expression of other Rap GEFs, especially PDZ-GEF1 (Fig. 6e). Taken together, these data indicate that the Rap GEF PDZ-GEF2 plays a major role in controlling cell spreading and phosphorylation of focal adhesion proteins downstream of Dsg2.

### Dsg2 harnesses Rap-induced TGFβ signaling to modulate effects on cell spreading

**Figure 7:**
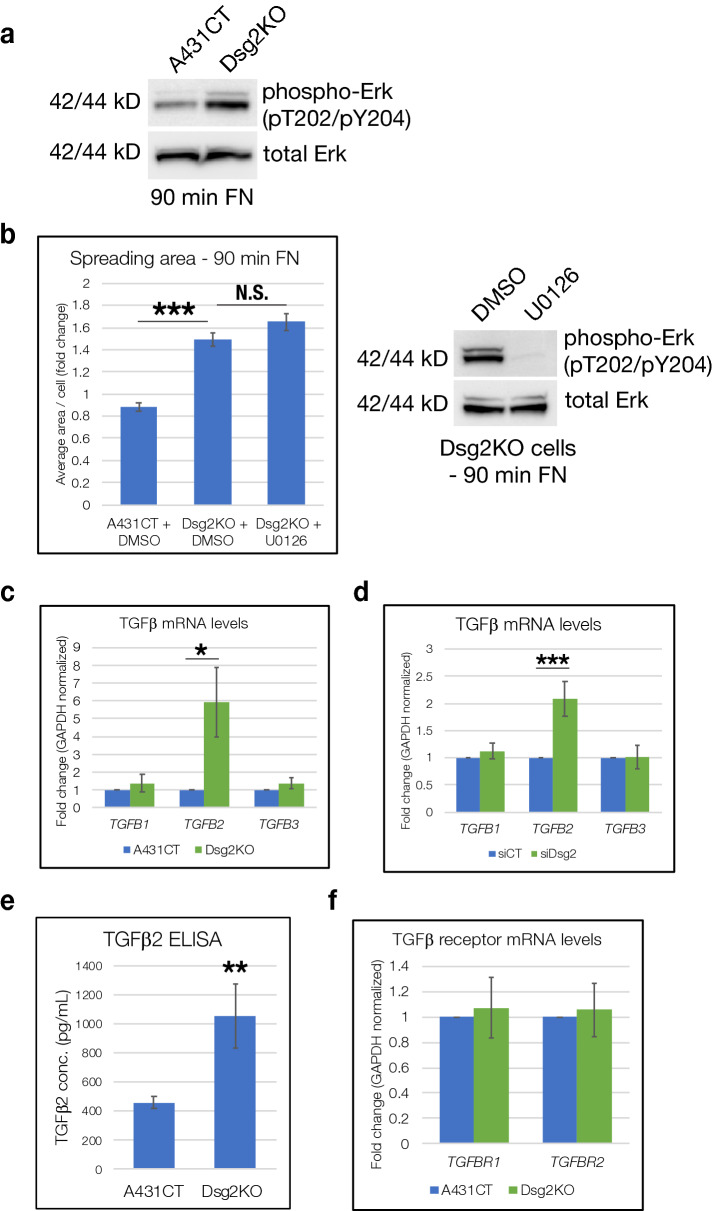
TGFβ and Erk signaling pathways are elevated in Dsg2KO cells compared to A431CT. (**a**) A431CT and Dsg2KO cells spreading on FN for 90 min were processed for SDS-PAGE and blotted with the following antibodies: phospho T202/Y204 Erk or total Erk. (**b**) Dsg2KO cells were pre-treated with U0126 for 30 min in suspension (or DMSO used as a vehicle control), plated on FN-coated coverslips for 90 min, and spreading area quantified. Graph represents average area/cell, with error bars indicating s.e.m. ***p < 0.001; N.S. = not significant. Loss of Erk activity upon treatment with U0126 was confirmed via western blot with phospho T202/Y204 Erk and total Erk. (**c**,**f**) A431CT & Dsg2KO cells were subjected to quantitative PCR to analyze mRNA levels of the following genes: TGFβ1 (*TGFB1*), TGFβ2 (*TGFB2*), TGFβ3 (*TGFB3*), TGFβ Receptor I (*TGFBR1*) and TGFβ Receptor 2 (*TGFBR2*). Graph represents fold change values normalized to GAPDH, with error bars indicating s.d. *p < 0.05. (**d**) siCT or siDsg2 transfected cells were subjected to quantitative PCR to analyze mRNA levels of the following genes: TGFβ1 (*TGFB1*), TGFβ2 (*TGFB2*) and TGFβ3 (*TGFB3*). Graph represents fold change values normalized to GAPDH, with error bars indicating s.d. ***p < 0.05. (**e**) Cell culture supernatants from A431CT & Dsg2KO cells were analyzed for protein levels of TGFβ2 via ELISA, as described in materials and methods. Graph represents changes in TGFβ2 concentration (pg/mL), with error bars indicating s.d. **p < 0.01. All unprocessed blots from this figure are shown in Supplementary Fig. S8.

To further delineate the mechanisms via which Dsg2-controlled alterations in Rap1 may affect cell spreading dynamics, we investigated signaling pathways known to be affected by Dsg2 and Rap1. Compared to A431CT cells, Dsg2KO cells demonstrated elevated phosphorylation of Erk (Fig. 7a). Nevertheless, abrogation of Erk signaling via the inhibitor U0126 did not rescue the increase in spreading area seen in Dsg2KO cells (Fig. 7b). These data indicate that while Erk signaling is elevated in Dsg2KO cells, it is not a causative factor in modulation of cell spreading via Dsg2.

Prior work has described changes in TGFβ signaling upon loss of desmosomal proteins such as PKP2 and DP^28^. Intriguingly, we observed a specific increase in TGFβ2 mRNA levels in Dsg2KO cells, whereas TGFβ1 and TGFβ3 mRNA levels remained unperturbed compared to A431CT cells (Fig. 7c). The specific increase in TGFβ2 mRNA levels (but not TGFβ1 or TGFβ3) was recapitulated upon siRNA-mediated knockdown of Dsg2, providing further validation of this finding (Fig. 7d). Secreted TGFβ2 protein levels (measured via ELISA analysis of cell culture supernatants) was also elevated in Dsg2KO cells compared to A431CT cells (Fig. 7e). mRNA levels of the two TGFβ receptors (*TGFBRI* *&* *TGFBRII*) remained unaffected in Dsg2KO cells, suggesting that changes in TGFβ2 mRNA levels are not a consequence of increased expression of TGFβ receptors (Fig. 7f).

**Figure 8:**
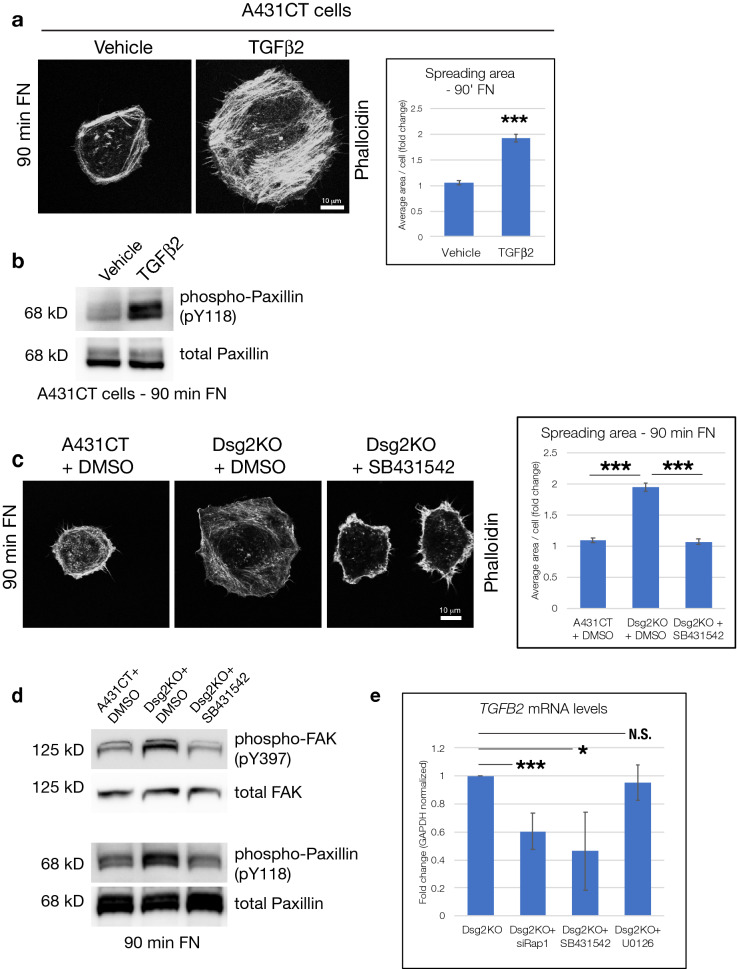
Dsg2 harnesses Rap-induced TGFβ signaling to modulate effects on cell spreading. (**a**) A431CT cells were treated with vehicle control or recombinant human TGFβ2 (20 ng/mL) during spreading for 90 min on FN, and spreading area quantified. Graph represents average area/cell, with error bars indicating s.e.m. ***p < 0.001. (**b**) A431CT cells treated with vehicle control or recombinant human TGFβ2 were processed for SDS-PAGE and blotted with the following antibodies: phospho-Paxillin (pY118) and total Paxillin. (**c**) A431CT + DMSO (vehicle), Dsg2KO + DMSO and Dsg2KO + SB431542 cells (pre-treated for 30 min in suspension) were plated on FN-coated coverslips for 90 min, and spreading area quantified. Graph represents average area/cell, with error bars indicating s.e.m. ***p < 0.001. (**d**) A431CT + DMSO (vehicle), Dsg2KO + DMSO and Dsg2KO + SB431542 treated cells were subjected to spreading on FN for 90 min, processed for SDS-PAGE and blotted with the following antibodies: phospho-Paxillin (pY118), total Paxillin, phospho-FAK (pY397) and total FAK. All unprocessed blots from this figure are shown in Supplementary Fig. S8. (**e**) Dsg2KO cells transfected with siRap1 or treated with either SB431542 or U0126 were subjected to quantitative PCR to analyze mRNA levels of TGFβ2 (*TGFB2*). Graphs represent fold change values normalized to GAPDH, with error bars indicating s.d. *p < 0.05, ***p < 0.001, N.S. = not significant.

To determine the role of TGFβ2 in cell spreading on FN, we treated A431CT cells with a vehicle control or recombinant human TGFβ2 during cell spreading (90 min FN). These experiments showed that treatment of A431CT cells with TGFβ2 alone is sufficient to produce an increase in cell spreading and paxillin phosphorylation (Fig. 8a,b). To analyze whether elevated TGFβ signaling is a causative factor in Dsg2-mediated cell spreading, we treated actively spreading cells with the TGFβ receptor inhibitor SB431542. As previously shown, Dsg2KO cells demonstrated a significant increase in cell spreading area compared to A431CT cells, and treatment with SB431542 was able to completely reverse this increase (Fig. 8c). SB431542 was also sufficient to rescue the elevated phosphorylation of both FAK and Paxillin seen in Dsg2KO cells (Fig. 8d). These data therefore clearly implicate TGFβ signaling in Dsg2-mediated cell spreading.

To identify whether Dsg2-mediated changes in Rap1 and TGFβ2 were part of the same signaling pathway or parallel signaling effects, we analyzed TGFβ2 mRNA levels upon Rap1 knockdown. Loss of Rap1 expression in Dsg2KO cells significantly reduced TGFβ2 mRNA levels, as did SB431542 inhibition (Fig. 8e). These data suggest that elevated TGFβ2 mRNA levels are a downstream consequence of enhanced Rap1 activity in Dsg2KO cells, and that high TGFβ2 mRNA levels are maintained via autocrine activation of the TGFβ receptor complex in Dsg2KO cells. In contrast to the significant effect observed with Rap1 siRNA, treatment with U0126 did not affect TGFβ2 mRNA levels in Dsg2KO cells, providing further evidence that Erk signaling is not involved in control of cell spreading via Dsg2 (Fig. 8e). Altogether, our work has demonstrated that loss of Dsg2 triggers PDZ-GEF2/Rap1 and TGFβ signaling which leads to increased phosphorylation of focal adhesion proteins and increased cell spreading on ECM.

## Discussion

As one of the major intercellular adhesive junctions, desmosomes have been well studied for their role in maintaining cell–cell adhesion and tissue integrity^1–3^. While they have historically been considered simple static “spot-welds”, studies in recent years have uncovered a multitude of different functions for desmosomal proteins in cellular processes as wide-ranging as proliferation, differentiation, gene expression, apoptosis, cell shape and migration^29^. These studies provide an updated picture of the desmosome as a complex of proteins with both structural and signaling roles. There are four Desmoglein isoforms (Dsg1–4) and three Desmocollin isoforms (Dsc1–3), whose expression varies depending on the type of tissue and stage of differentiation^4^. In connection with their primary adhesive role, desmosomal cadherins are well known for their regulation of epidermal morphogenesis and differentiation^7^.

Dsg2 is one of the most widely expressed Desmogleins found in simple epithelia, complex epithelia (basal layer) and the myocardium^30^. Dsg2 null embryos die at implantation, likely due to defects in proliferation of Dsg2-deficient embryonic stem cells^31^. Mutation and loss of Dsg2 has also been linked to arrhythmogenic cardiomyopathy, a genetic condition characterized by loss of strong cell–cell adhesion and elevated fibrotic gene expression in cardiomyocytes^32^. Changes in expression of desmosomal cadherins (from Dsg2 in undifferentiated basal keratinocytes to Dsg1 in suprabasal differentiated keratinocytes) is critically important for coordinating the process of keratinocyte differentiation and maintaining the structure and function of the epidermis^33^. Dsg1 in particular is important for promoting differentiation by suppressing Epidermal Growth Factor Receptor (EGFR) and Erk signaling during the process of epidermal differentiation. Intriguingly, Dsg1 can modulate its effects on keratinocyte differentiation without its ectodomain, indicating that engagement of the Dsg1 extracellular domains is not a pre-requisite for this function^13^.

A role for desmosomal cadherins in the process of cell migration has also been extensively studied, usually in the context of collective cell migration^29^. Different isoforms of Desmoglein and Desmocollin have been ascribed both pro- and anti-migratory functions, suggesting that their effects are not simply a consequence of loss of cell–cell adhesion, but instead contribute complex cell type-specific signaling roles to the processes of cell migration and metastasis^34–42^. Overexpression of Dsg3 increases the rate of migration and formation of filopodia, likely through downstream regulation of Src^34^. Another study showed that overexpression of Dsg3 results in an increase in Rac1 and Cdc42 activity and formation of lamellipodia/filopodia^35^. Ectopic expression of Dsg3 has also been shown to induce PKC-dependent phosphorylation of the actin binding protein Ezrin, leading to elevated migration and invasion of different cancer cell lines^36^. In contrast to these studies describing pro-migratory effects of Dsg3, loss of Dsg3 in keratinocytes was shown to promote cell migration via p38MAPK signaling^37^. Several studies have also described anti-migratory functions for Dsg2. Silencing of Dsg2 results in enhanced migration through deregulated EGFR/Erk signaling^38^. Dsg2 depletion also increases the expression of migration-related genes such as secretogranin II, which enhanced the migratory activity of melanoma cells^39^. Lastly, loss of Desmocollins (Dsc2 or Dsc3) has also generally been associated with enhanced migration and metastasis^40–42^.

As seen above, roles for desmosomal proteins in mediating cell migration is well studied, but how these proteins mediate ECM attachment and cell spreading is less well understood. In addition, only a few studies have investigated whether desmosomal proteins can mediate effects on cell–matrix adhesion and spreading in a cell autonomous or junction-independent manner. Our previous work has described a role for DP in mediating actin cytoskeletal dynamics via Rac1 and p38 MAPK signaling, an effect which is maintained when analyzing single spreading cells^15^. PKP2 has also been shown to control single cell spreading, where loss of PKP2 results in decreased cell spreading and decreased phospho-Paxillin containing focal adhesions^14^. While these studies describe non-junctional functions for the cytoplasmic plaque proteins DP and PKP2, a more intriguing question is whether transmembrane desmosomal cadherins can mediate effects on cell–matrix adhesion and spreading, and whether this can occur in the absence of cell–cell contact. Our results show that loss of Dsg2 does induce changes in single cell spreading, and that this effect is the opposite of loss of PKP2, with Dsg2KO cells demonstrating enhanced spreading area and increased phosphorylation of focal adhesion proteins (Figs. 2, 3). Moreover, these results are specific to Dsg2, as loss of the other commonly expressed desmosomal cadherin Dsc2 did not recapitulate the same effect on cell spreading (Fig. 4).

Attachment to the ECM via focal adhesions is achieved via transmembrane integrin complexes (α and β integrin heterodimers), which serve as a bridge between ECM proteins and a wide range of intracellular adaptor and signaling proteins, some of which link to the actin cytoskeleton^16,43^. Integrin-mediated adhesion to the ECM results in rapid phosphorylation and activation of FAK and Paxillin, both of which signal downstream to a range of different GTPases involved in coordinating actin cytoskeletal changes needed for efficient spreading and migration^16^. Our findings report a significant increase in phosphorylation of the focal adhesion proteins FAK and Paxillin during active cell spreading (90 min FN), but no change in expression of all α and β integrins tested, suggesting that Dsg2 controls cell spreading via signaling to focal adhesion proteins but not changes in integrin expression (Fig. 3).

Intriguingly, the ability of Dsg2 to modulate changes in focal adhesion protein phosphorylation is lost in confluent monolayers of cells (Fig. 3). We hypothesize that the inability of Dsg2 to affect focal adhesion phosphorylation in the presence of uniform, widespread cell–cell junctions is an indication that signaling mechanisms mediated by other junctional proteins may mask or abrogate the effects of Dsg2 seen in singly spreading cells (where intercellular connections are non-existent). Our results from cell spreading assays therefore show that Dsg2 has a cryptic role in mediating cell attachment and spreading completely independent of cell–cell adhesion, thereby implicating Dsg2 in the modulation of biological processes where the behavior of single cells or small cell clusters is highly relevant, such as wound healing and metastatic dissemination.

The Rap1 GTPase has emerged as a central player in ECM attachment and spreading, being known to coordinate both outside-in signaling (from ECM proteins to intracellular signaling pathways) and inside-out signaling (to modulate activity of integrins and focal adhesion proteins). Loss of Rap1 has been shown to cause reduced activation of integrins and reduced cell spreading, whereas overexpression of Rap1 has the opposite effect, clearly highlighting a role for Rap1 in promotion of integrin-mediated adhesion^17,44^. Our results show that Rap1 activity is elevated in Dsg2KO cells, and knockdown or inhibition of Rap1 can rescue the enhanced spreading seen in Dsg2KO cells, as well as the enhanced phosphorylation of focal adhesion proteins (Fig. 5). These data therefore point to a central mechanistic role for Rap1 in Dsg2-mediated control of cell spreading dynamics.

Several previous studies have analyzed signaling connections between desmosomal proteins and Rap1. The armadillo protein PKP3 forms a complex with Rap1 and enhances its activity, which is required for proper assembly of desmosomes and adherens junctions^45^. A recent study has also shown that loss of Dsc2 results in reduced Rap1 activity and decreased cell migration, effects which may be a consequence of loss of PKP3 expression in cells lacking Dsc2^26^. In our study, we found no change in PKP3 expression upon Dsg2 knockout (Fig. 1). Further, our results show that loss of Dsg2 elevates Rap1 activity (the opposite effect shown by Dsc2 in the above study), suggesting that these different desmosomal cadherins may control Rap1 activity via distinct signaling pathways. To our knowledge, our study is the first to demonstrate the ability of Dsg2 to control Rap1 signaling during cell spreading.

To analyze the possible mechanisms via which Dsg2 may control Rap1 activity, we investigated different Rap1 regulators, several of which have been implicated in cell–matrix adhesion and spreading. Overexpression of Rap1GAP causes a reduction in β1 integrin activation, phosphorylation of FAK and Paxillin and decreased cell spreading, while knockdown of Rap1GAP has opposing effects^17,46,47^. Previous reports have also implicated a variety of different Rap GEFs such as C3G, Epac1/2 and PDZ-GEFs in Rap-mediated control of cell spreading^19^. We therefore sought to determine which Rap GEFs are involved in mediating the effects of Dsg2 on cell spreading. Using siRNA-mediated knockdown of individual Rap GEFs (Epac1, Epac2, C3G, PDZ-GEF1 or PDZ-GEF2), we demonstrate that loss of PDZ-GEF2 had the most significant impact on cell spreading and focal adhesion protein phosphorylation in Dsg2KO cells (Fig. 6). Our results therefore indicate that Dsg2 requires the Rap GEF PDZ-GEF2 to modulate its effects on cell spreading and corroborate previous reports describing a positive effect of PDZ-GEF2 on integrin-mediated adhesion via Rap1^27,48^.

We next sought to investigate the involvement of other signaling proteins commonly known to be downstream of both Dsg2 and Rap1. Loss of Dsg2 was previously shown to induce elevated Erk activity, inhibition of which could rescue the enhanced migration of Dsg2-deficient cells^38^. Rap has also been shown to elevate Erk activity in many different cell types^49,50^. Based on these reports, we were not surprised to find in our study that Dsg2KO cells (which demonstrate elevated Rap1 activity) also have increased levels of phosphorylated (active) Erk. Nevertheless, inhibition of Erk signaling via U0126 treatment did not rescue the enhanced cell spreading seen in Dsg2KO cells. These data indicate that although Erk activity is elevated in Dsg2KO cells, it does not play a causative role in mediating cell spreading dynamics downstream of Dsg2.

Lastly, we investigated a potential role for TGFβ in Dsg2/Rap1-mediated cell spreading, as TGFβ signaling has been shown to be elevated upon loss of other desmosomal proteins. Specifically, knockdown of either PKP2 or DP in neonatal cardiomyocytes results in elevation of TGFβ1 mRNA, but not TGFβ2 or TGFβ3^28^. In contrast to loss of PKP2 or DP, we found that either Dsg2 knockout or knockdown resulted in elevation of TGFβ2 mRNA, but not TGFβ1 or TGFβ3 (Fig. 7). In addition, inhibition of TGFβ signaling via SB431542 was able to rescue both the enhanced cell spreading and elevated phosphorylation of FAK and Paxillin seen in Dsg2KO cells. The effect on TGFβ2 mRNA expression is downstream of Dsg2-mediated control of Rap1, as Rap1 knockdown was sufficient to decrease TGFβ2 mRNA levels in Dsg2KO cells (Fig. 8). Although Rap1 has previously been shown to promote TGFβ signaling via enhanced transcription of TGFβ receptor II (*TGFBR2*)^51^, this does not appear to be the mechanism involved here, as we see no changes in mRNA levels of either TGFβ receptor (*TGFBRI* or *TGFBRII*) between A431CT and Dsg2KO cells.

Further questions remain related to the role of other desmosomal proteins in Dsg2-mediated cell spreading. Compared to A431CT cells, Dsg2KO cells showed no dramatic differences in the distribution of DP, PKP2 or PG during spreading of cells for 90 min on FN (Supplementary Fig. S1). Total protein levels of DP, PG and PKP2 are also unaffected by Dsg2KO in actively spreading cells (Fig. 2e). Taken together, these results suggest that Dsg2 does not exert dramatic effects on overall expression and localization of other desmosomal proteins during single cell spreading, which is in contrast to the important role Dsg2 plays in anchoring and stabilizing desmosomal proteins at the junctional plaque in confluent monolayers of cells^52^. Nevertheless, we do not believe these results rule out a role for other desmosomal proteins in mediating the effects of Dsg2 on cell spreading, as more subtle changes in cytoskeletal linkage or protein interactions with other signaling molecules may underlie important roles for other desmosomal proteins in mediating cell spreading dynamics via Dsg2, and these possibilities merit further scrutiny.

In summary, our work has identified a novel function for Dsg2 in dampening cell spreading and phosphorylation of focal adhesion proteins. Importantly, these effects occur in a cell autonomous fashion, indicating that Dsg2 can control cell spreading independent of desmosome-mediated cell–cell contact. Dsg2-mediated control of cell spreading is a consequence of PDZ-GEF2/Rap1 and TGFβ signaling, both of which are involved in activation of focal adhesion proteins which orchestrate cell attachment and spreading. Our findings highlight the importance of investigating signaling connections between desmosomes and cell–matrix attachments and add to the body of evidence which describe non-junctional functions for proteins of the desmosome.

## Methods

### Cell culture, siRNA transfections and drug treatments

A431 cells were cultured in DMEM with 10% Fetal Bovine Serum and antibiotic/antimycotic solution (Corning). HaCaT keratinocytes were cultured in low calcium (0.05 mM) DMEM (to maintain them in a proliferative, undifferentiated state), 10% chelexed Fetal Bovine Serum and antibiotic/antimycotic solution (Corning). Dsg2 knockout A431 cells (Dsg2KO) were generated via CRISPR/Cas9-mediated targeting and cleavage as described in Ref.^23^. For siRNA-mediated knockdown, cells at ~ 30–40% confluency were transfected with either control or gene-specific siRNA (Integrated DNA Technologies) using DharmaFECT1 (Dharmacon) according to manufacturer’s protocols, followed by harvesting cells for subsequent analysis at 72 h post siRNA transfection. Please see Supplementary Table S1 for all siRNA target sequences used in this study. Where indicated, A431 cells were treated with the following drugs purchased from MilliporeSigma: U0126 (20 µM), SB431542 (5 µM) or GGT1-298 (10 µM). For TGFβ2 stimulation, A431CT cells were treated with a vehicle control (4 mM HCl + 0.1% bovine serum albumin in sterile water) or recombinant human TGFβ2 (20 ng/mL) from R&D Systems.

### Cell spreading assays

For cell spreading assays, coverslips in 12 well plates were pre-coated with either 30 µg/mL human fibronectin or rat tail type I collagen (Corning) for 1 h at 37 °C. To plate cells on pre-coated coverslips, cells were trypsinized, pelleted and resuspended in DMEM + 0.1% Bovine Serum Albumin. Cells were held in suspension for 30 min via gentle rotation at 37 °C, followed by plating on to the pre-coated coverslips at a very low density to allow for single cell spreading. Following spreading on ECM proteins for the times indicated, the coverslips were fixed and processed for immunofluorescence, as described in detail in the sections below. Imaging of single cell spreading was performed following F-actin staining or pre-treatment with CellTracker Green CMFDA (5-chloromethylfluorescein diacetate), purchased from ThermoFisher Scientific (0.5 μM CMFDA added 30 min prior to plating on FN). Quantification of cell spreading area is described in detail in the section below.

### Immunofluorescence, microscopy and quantification of images

Cells on coverslips were fixed in 4% paraformaldehyde (Electron Microscopy Sciences) for 15–30 min, washed 3× with Phosphate Buffered Saline (PBS), permeabilized with 0.3% Triton X-100 for 10 min (MilliporeSigma), and blocked with 5% normal donkey serum (Jackson Immunoresearch) for 60 min at 37 °C. Coverslips were incubated with AlexaFluor 568 Phalloidin (ThermoFisher) or primary and secondary antibodies (listed in Supplementary Table S2) and mounted in Prolong Gold + DAPI (ThermoFisher). All immunofluorescence images shown were taken using a Leica Microsystems TCS SP8 Spectral Confocal Microscope, an oil immersion 63× objective (HC PL APO 63×/1.40 OIL CS2) and Leica LAS × SP8 control software. All images shown are representative of three or more experiments. For quantification of cell spreading, 10–30 random fields of view were imaged per sample using the same exposure time and camera settings. Spreading area per cell was quantified using ImageJ software (NIH), and statistical analysis performed as described below. Thresholding is applied to each image to segment stained cells from the background image, followed by use of the outline tool to highlight and measure cell area. This process is repeated in 10–30 randomly imaged areas of each experimental sample till area measurements have been recorded for a minimum of 50–200 cells per sample. Average area/cell is plotted on graphs and represented as fold change (i.e., percent change from control cells), as has been done in several prior reports investigating changes in cell spreading^53–55^. Cell diameter and protrusiveness (measured as distance from nucleus to edge of lamellipodial protrusions) was quantified using the line tool in ImageJ, as we have done previously^15^.

### Western blots and Rap1-GTP pulldowns

To analyze protein expression levels, cells were washed briefly in phosphate buffered saline (PBS), lysed in urea sample buffer (8 M deionized urea, 1% SDS, 10% Glycerol, 60 mM Tris pH 6.8, and 5% β-mercaptoethanol) and equalized for total protein concentration using a Take3 plate and a Synergy LX plate reader (Biotek). Samples were subjected to SDS-PAGE using Bis–Tris SurePAGE precast polyacrylamide gels (Genscript), followed by transfer to PVDF membranes (MilliporeSigma). Membranes were probed with primary and secondary antibodies (listed in Supplementary Table S2), washed in Tris Buffered Saline + 0.2% Tween and blots visualized by enhanced chemiluminescence using a ChemiDoc Imaging System (BioRad). In order to blot for several proteins of different molecular weights, membranes were frequently cut into different segments using the molecular weight ladder as a guide, and individual segments probed with different antibodies. For example, this approach allowed us to confirm equal loading of all gels by probing for GAPDH on all membranes. Unprocessed images obtained from the BioRad ChemiDoc Imaging System for all blot segments shown in this manuscript have been included in Supplementary Figs. S3–S8. All western blots shown are representative of data from three or more independent experiments. To measure Rap1 activity, purified GST-Ral GDS Rap Binding Domain (RBD) bound to agarose beads was used to pulldown GTP-bound Rap1 from cellular lysates, according to manufacturer’s protocols (MilliporeSigma). Pulldown and total lysate samples collected in Laemmli buffer with 5% β-mercaptoethanol were subjected to SDS-PAGE and blotted with an anti-Rap1A/B antibody (Cell Signaling Technology). For quantification of changes in protein levels, densitometric analysis was performed via ImageJ software. Pixel intensity of bands was measured from three independent experiments, normalized to a reference (GAPDH, or total protein levels for phosphorylated focal adhesion proteins and Rap1-GTP levels), and statistical analysis performed as described below.

### TGFβ2 ELISA

To measure levels of TGFβ2 in cell culture supernatant of A431CT and Dsg2KO cells, a Quantakine TGFβ2 ELISA (R&D Systems) was performed according to manufacturer’s instructions. Briefly, quadruple samples of cell culture supernatant of A431CT and Dsg2KO cells were cleared of cell debris by brief centrifugation, followed by activation of latent TGFβ2 to the immunoreactive form by 10 min of acid treatment and subsequent neutralization. Activated A431CT and Dsg2KO cell culture supernatant samples were added to wells pre-coated with a monoclonal antibody specific for human TGFβ2 (along with a series dilution of human TGFβ2 standards), and incubated for 2 h at room temperature. Following washes, the wells are incubated with an enzyme-linked polyclonal TGFβ2 antibody for 2 h at room temperature. Levels of TGFβ2 are calculated via addition of substrate and measuring absorbance of the colored product at 450 nm, and statistical analysis was performed as described below.

### Quantitative real-time PCR

To measure mRNA transcript levels via quantitative real-time PCR (qPCR), RNA was isolated using the RNeasy Mini kit, according to manufacturer’s instructions (Qiagen). Total RNA concentrations were equalized between samples using a Nanodrop spectrophotometer (ThermoFisher), and qPCR was performed using gene-specific primers and Power SYBR Green RNA-to-C_T_ 1-step kit (Applied Biosystems). qPCR cycling was performed in a QuantStudio3 instrument (Applied Biosystems). All gene-specific primers used for qPCR in this study are listed in Supplementary Table S3. Calculations for relative mRNA levels were performed using the ΔΔC_T_ method, normalized to GAPDH, and represented as fold change values compared with control samples. All graphs for mRNA levels shown were obtained from quantification of three or more independent biological replicates, and statistical analysis was performed as described below.

### Statistical analysis

For all experiments shown, data was obtained from a minimum of three independent experiments/biological replicates. In many cases, multiple technical replicates (wells, coverslips, etc.) were processed in parallel within each independent experiment. Representative images/blots are displayed in all figures, and graphs for each experiment are indicated in the figure legends as either mean ± standard deviation (s.d.) or mean ± standard error of the mean (s.e.m.). For cell area measurements, 50–200 cells were counted for each condition from 10 to 30 randomly imaged areas. All statistical analysis was performed using SPSS Statistics software (IBM). For data comparing two conditions (A431CT vs. Dsg2KO), statistical analysis was performed using a student’s two-tailed *t* test. For data comparing three or more conditions, statistical analysis was performed using Welch’s ANOVA followed by Games–Howell post hoc analysis. Statistical significance was represented in graphs as follows: N.S. = not significant, *p < 0.05, **p < 0.01, ***p < 0.001.

## Supplementary Information

Supplementary Information.

## Data Availability

All data generated or analyzed for this study are included in this published article (and its Supplementary Information files).

## Abbreviations

Dsg2: Desmoglein-2
PDZ-GEF2: PDZ Guanine Nucleotide Exchange Factor 2
Rap1: Ras proximate 1 GTPase
TGFβ: Transforming growth factor beta
FAK: Focal adhesion kinase
ECM: Extracellular matrix
FN: Fibronectin
