# Supplementary material for: Desmoglein-2 harnesses a PDZ-GEF2/Rap1 signaling axis to control cell spreading and focal adhesions independent of cell–cell adhesion
Source: Sci Rep. 2021 Jun 24;11:13295. doi: 10.1038/s41598-021-92675-1 (PMC8225821; doi:10.1038/s41598-021-92675-1)

SUPPLEMENTARY INFORMATION

Desmoglein-2 harnesses a PDZ-GEF2 / Rap1 signaling axis to control cell spreading and focal adhesions independent of cell-cell adhesion.

W. Tucker Shelton[1], S. Madison Thomas[1], Hunter R. Alexander[1], C. Evan Thomes[1], Daniel E. Conway[2], Adi D. Dubash[1*].

[1]Furman University, Department of Biology, 3300 Poinsett Highway, Greenville SC 29613, USA.
[2]Virginia Commonwealth University, Department of Biomedical Engineering, 601 West Main Street, Richmond VA 23284, USA.

Address correspondence to:
Adi D. Dubash, Ph.D.
Department of Biology, Furman University
adi.dubash@furman.edu

Included in Supplementary Information:
Supplementary Tables S1-3.
Supplementary Figures S1-8.

SUPPLEMENTARY TABLES

Supplementary Table S1: Gene-specific siRNA target sequences

For siRNA-mediated knockdown, the following gene-specific siRNA was used (purchased from Integrated DNA Technologies). The protocol used for transfection of siRNA is included in the methods section.

Gene	siRNA target sequence
DSG2	CUCAGCAUCUUCAAGAUGUACCUTA
DSC2	GUGAGAGAUAGACUUGGCAUGUCTA
RAP1A	ACAUCAUGCGUGAGUACAAGCUAGT
RAP1B	UAUUUAAAUUGACCAACCUAAUGTT
RAPGEF1 (C3G)	GGUCUUGUUGCCAAUUUACAAACCG
RAPGEF2 (PDZ-GEF1)	GGUCAAUUUUGAGAAGCUAAGGATG
RAPGEF3 (Epac1)	GAAAUUGUUAUGGUACAUGAGCATC
RAPGEF4 (Epac2)	CAAGCAGGACUUCAACCGUAUCATC
RAPGEF6 (PDZ-GEF2)	GGAUCAGUGAAUGUAGUCAUUUACG

Supplementary Table S2: Primary and Secondary Antibodies

The source of all primary and secondary antibodies used in this study are listed here, as well as molecular weights (in kiloDaltons) and dilutions used for western blots (WB) or immunofluorescence (IF). The protocols used for immunofluorescence and western blotting with these antibodies are detailed in the methods section.

Antibody	Source/Vendor	Catalog Number	Dilution	Molecular weight (kDa)
anti-Desmoglein 2 (6D8)	ThermoFisher	32-6100	WB 1:500	140
anti-Desmocollin 2/3 (7G6)	ThermoFisher	32-6200	WB 1:500	120
anti-Desmoglein 3 (5G11)	ThermoFisher	32-6300	WB 1:500	130
anti-E-Cadherin (HECD-1)	Abcam	ab1416	WB 1:1000	135
anti-Desmoplakin (NW6)	Kathleen Green, Northwestern University	N/A	WB 1:1000	240/260
anti-pan-Keratin (C11)	Cell Signaling Technology	4545	WB 1:1000	45/55
anti-Plakophilin-2 (C-1)	Santacruz Biotechnology	sc-393711	WB 1:200	100
anti-Plakophilin-3 (E-10)	Santacruz Biotechnology	sc-166655	WB 1:200	90
anti-Plakoglobin (1407)	Kathleen Green, Northwestern University	N/A	WB 1:2000	80
anti-β-catenin (D10A8)	Cell Signaling Technology	8480	WB 1:2000	90
anti-p120-catenin	BD BioSciences	610134	WB 1:2000	120
anti-GAPDH (0411)	Santacruz Biotechnology	sc-365062	WB 1:1000	37
anti-phospho Y118 Paxillin (E9U9F)	Cell Signaling Technology	69363	WB 1:1000 / IF 1:500	68
anti-Paxillin	BD Biosciences	612405	WB 1:1000 / IF 1:500	68
anti-phospho Y397 FAK	BD Biosciences	611722	WB 1:500	125
anti-FAK	BD Biosciences	610087	WB 1:1000	125
anti-phospho Y416 Src (D49G4)	Cell Signaling Technology	6943	WB 1:500	60
anti-Src (36D10)	Cell Signaling Technology	2109	WB 1:500	60
anti-β1 integrin (D2E5)	Cell Signaling Technology	9699	WB 1:1000	135
anti-β4 integrin (D8P6C)	Cell Signaling Technology	14803	WB 1:1000	210
anti-Vinculin (hVin1)	MilliporeSigma	V9131	WB 1:2000	120
anti-Rap1A/B (26B4)	Cell Signaling Technology	2399	WB 1:500	21
anti-phospho tyrosine (PY99)	Santacruz Biotechnology	sc-7020	IF 1:200	N/A
anti-PDZ-GEF2	ThermoFisher	A301-967A	WB 1:500	210
anti-Erk1/2 (C-9)	Santacruz Biotechnology	sc-514302	WB 1:1000	42/44
anti-phospho T202/Y204 Erk1/2 (D13.14.4E)	Cell Signaling Technology	4370	WB 1:1000	42/44
Peroxidase-conjugated AffiniPure goat anti-mouse IgG	Jackson ImmunoResearch	115-035-146	WB 1:10,000	N/A
Peroxidase-conjugated AffiniPure goat anti-rabbit IgG	Jackson ImmunoResearch	111-035-144	WB 1:10,000	N/A
Peroxidase-conjugated AffiniPure goat anti-chicken IgG	Jackson ImmunoResearch	103-035-155	WB 1:10,000	N/A
AlexaFluor 488-conjugated AffiniPure donkey anti-mouse IgG	Jackson ImmunoResearch	715-545-151	IF 1:400	N/A
AlexaFluor 647-conjugated AffiniPure donkey anti-mouse IgG	Jackson ImmunoResearch	715-605-151	IF 1:400	N/A
AlexaFluor 488-conjugated AffiniPure donkey anti-rabbit IgG	Jackson ImmunoResearch	711-545-152	IF 1:400	N/A

Supplementary Table S3: Gene-specific forward and reverse qPCR primers

For quantitative real-time PCR (qPCR), the following gene-specific forward and reverse primers were used (purchased from Integrated DNA Technologies). The protocol used for qPCR is included in the methods section.

Gene	Forward Primer	Reverse Primer
DSC2	ATCCTGTTTACGCTGGTCTG	GTTGTGAAGCCATTCGCAG
DSG3	CGAATCTCTGGAGTGGGAATC	AGTCCTTGGGCATTTAGAGC
CDH1	CCCAATACATCTCCCTTCACAG	CCACCTCTAAGGCCATCTTTG
DSP	ACCAGAACCAGAACACCATC	GGGCAAAACACTCATCCAATTC
PKP2	TGATGGGAGAAAAGCGATGAG	GCTGGTAGGAGAGGTTATGAAG
PKP3	AACCTCATCTACGACAACGC	TGGTCGCTGGATGAAAGG
JUP	CTCTGTGCGTCTCAACTATGG	AGATTCCTGATCAAGCCGATG
CTNNB1	GTTCAGTTGCTTGTTCGTGC	GTTGTGAACATCCCGAGCTAG
CTNND1	GCGGGAACCTAATGAAGACTG	GGGCATCAACTAAACCATCAC
PXN	CTCAGGACAGTGTTGGCTC	CGGTCGAGTTCAGAAAGGTTG
FAK	AAATACGGCGATCATACTGGG	TTGGCCTTGACAGAATCCAG
ITGA1	AGGATTTCTGGCTTGTGGG	ACTATGTCCAGTTGAGTGCTG
ITGA3	GGAACAGCACCTTCATCGAG	AATGTCCACAGAGAACCACG
ITGB1	CCCAGAGGCTCCAAAGATATAAA	GCTGTGGTTGGATCTGAGTAA
ITGB4	AACGATGACAACCGACCTATT	GACTCCCGAAGGTTCTCAATAA
ITGB5	GTCTGCTAATCCACCCAAAATG	TCTCTATCTCACCTCCACAGC
TGFB1	GCCTTTCCTGCTTCTCATGG	TCCTTGCGGAAGTCAATGTAC
TGFB2	CCCCACATCTCCTGCTAATG	ATGTAAAGTGGACGTAGGCAG
TGFB3	AGTGGCTGTCCTTTGATGTC	CACCTCGTGAATGTTTTCCAG
TGFBR1	GCCAAATATCCCAAACAGATGG	ATGCCTTCCTGTTGACTGAG
TGFBR2	CTGTGGATGACCTGGCTAAC	CATTTCCCAGAGCACCAGAG
RAPGEF1	TGGGTGATTTTACTGCTCCTG	GTGGCGTCTGGTAGAACATAG
RAPGEF2	ATAAACCGAACGAGTCTGACC	TTCTTGTGCTCTCCTTTCAGG
RAPGEF3	CTCCATGCTCCACACTGAC	ATTCCCACAGCCATTCTCC
RAPGEF4	AAGGACTTCAAGGCACTATGG	AACGTGAGGTTCAATGAGAGG
RAPGEF6	ACAGAAAACCCTCATCCACAG	CTACATCTCCTACCTCACTCTCC
GAPDH	ACATCGCTCAGACACCATG	TGTAGTTGAGGTCAATGAAGGG

SUPPLEMENTARY FIGURE LEGENDS

Supplementary Figure S1: **(a)** Control A431 cells (A431CT) and Desmoglein-2 knockout cells (Dsg2KO) were trypsinized and held in suspension for 30 min during pre-treatment with CellTracker Green CMFDA (0.5 μM), followed by plating on FN-coated coverslips for 90-135 min. Coverslips were fixed and cell imaging and spreading area quantification performed as described in materials and methods. Graph represents fold change differences in average area/cell, with error bars indicating s.e.m. *** = $p < 0.001$ vs. control. **(b)** Control A431 cells (A431CT) and Desmoglein-2 knockout cells (Dsg2KO) were subjected to cell spreading on FN-coated coverslips for 90 min, followed by fixation and immunofluorescence for PKP2, PG and DP.

Supplementary Figure S2: **(a)** Control A431 cells (A431CT) and Desmoglein-2 knockout cells (Dsg2KO) were plated on FN-coated coverslips at low density. 24 hours after plating, coverslips were fixed and immunofluorescence performed for DAPI and total Paxillin. F-actin stained images of sub-confluent A431CT and Dsg2KO cells plated on FN for 24 h were used for spreading area quantification performed as described in materials and methods. Graph represents fold change differences in average area/cell, with error bars indicating s.e.m. *** = $p < 0.001$ vs. control. **(b)** Sub-confluent A431CT and Dsg2KO cells plated on FN for 24 h were subjected to SDS-PAGE and blotted for total Paxillin and phospho-Paxillin (pY118).

Supplementary Figure S1, Shelton *et. al.*

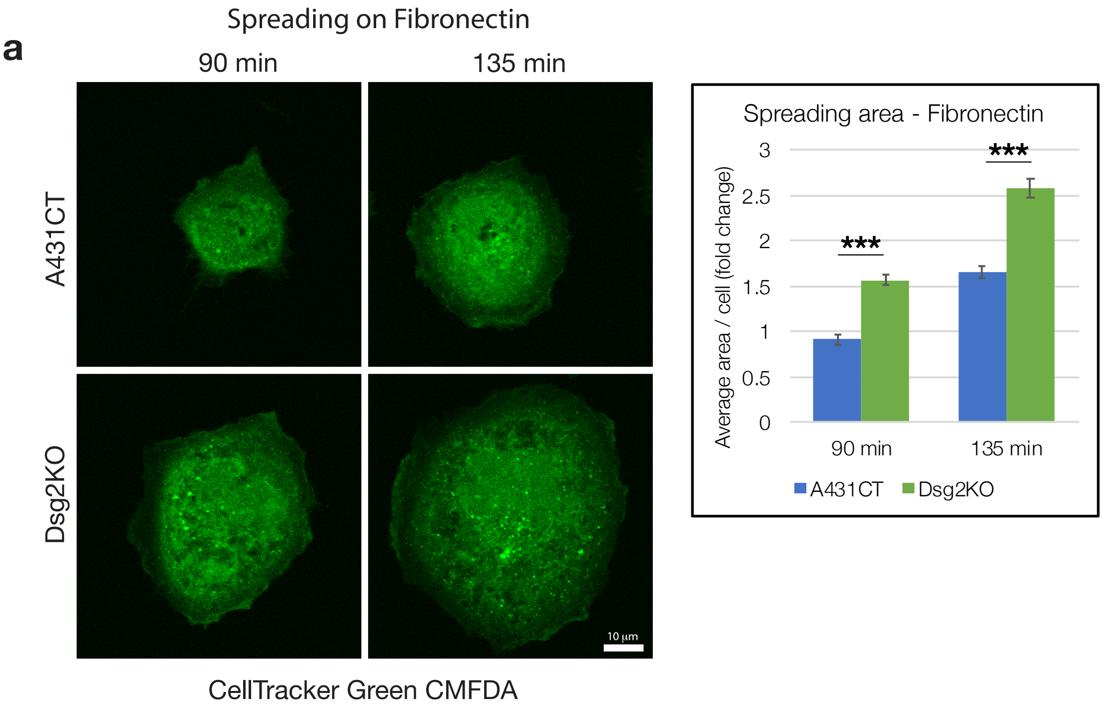

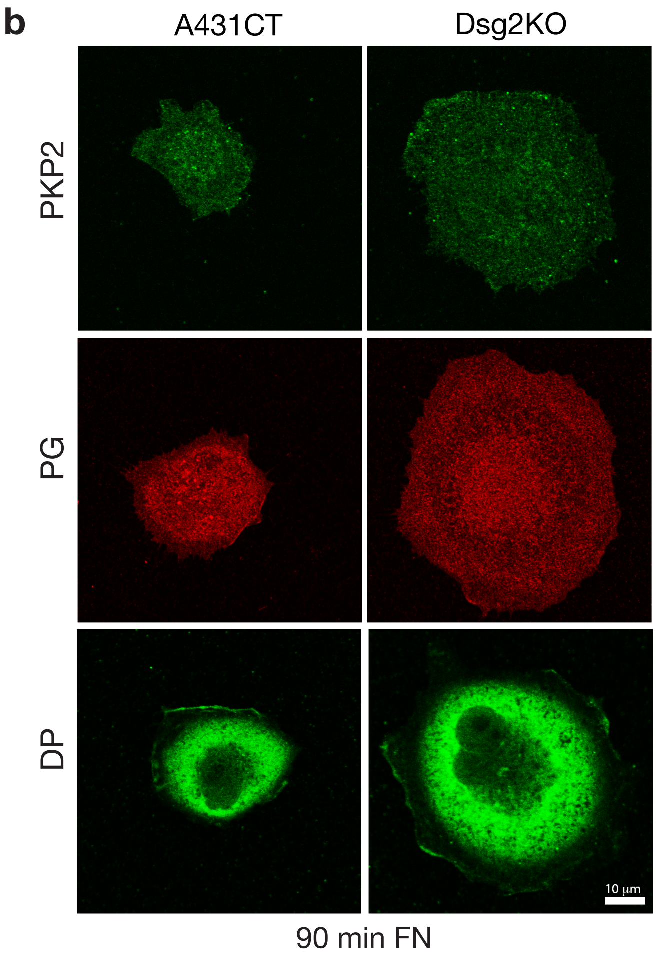

Supplementary Figure S2, Shelton *et. al.*

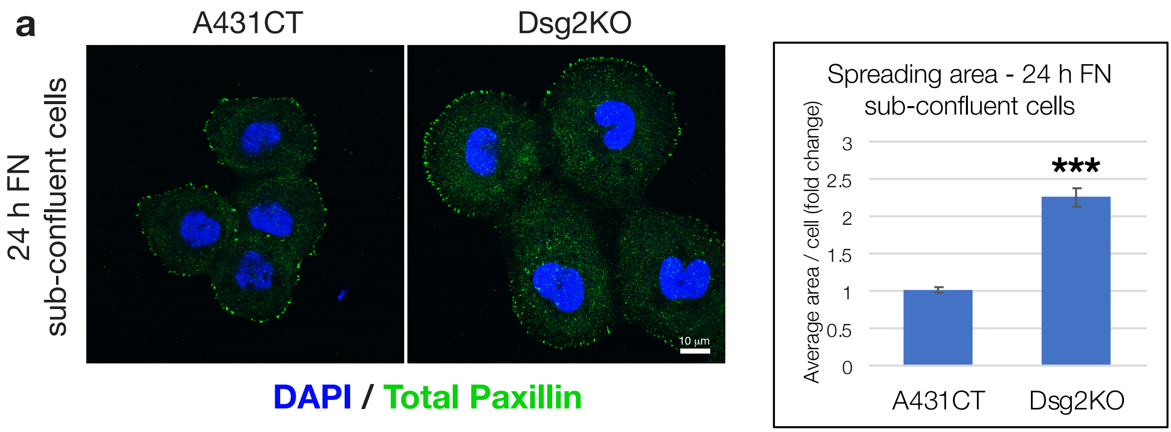

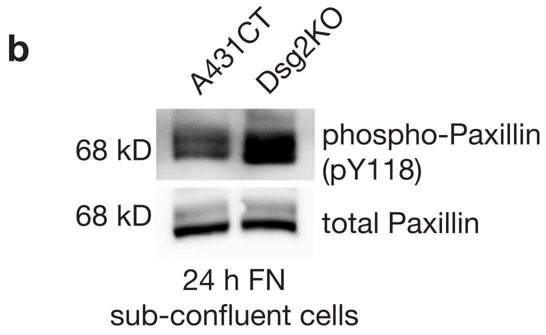

Supplementary Figure S3, Shelton WT *et. al.*

Unprocessed blot Images - Figure 1b

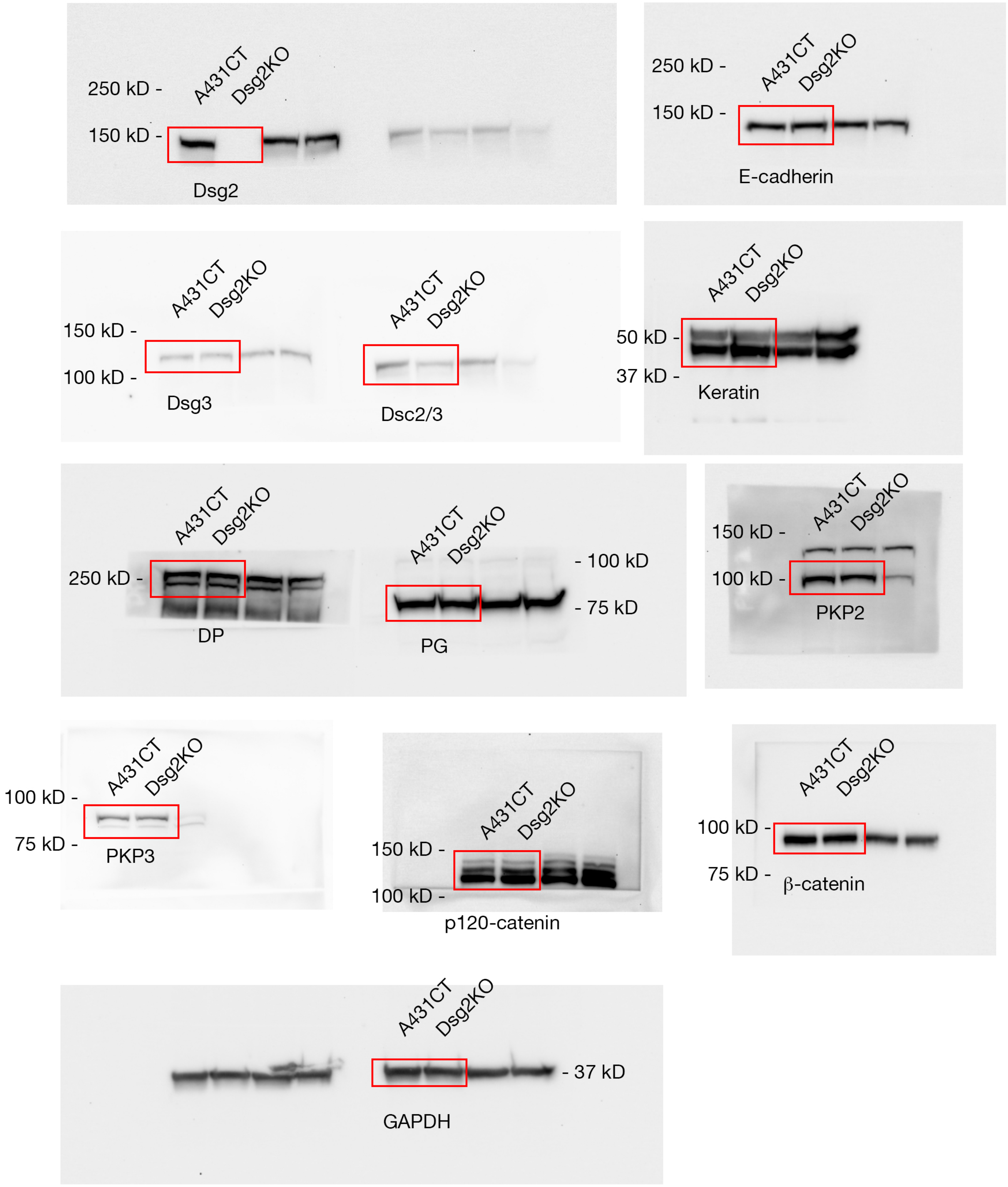

Supplementary Figure S4, Shelton WT *et. al.*

Unprocessed blot Images - Figure 2e

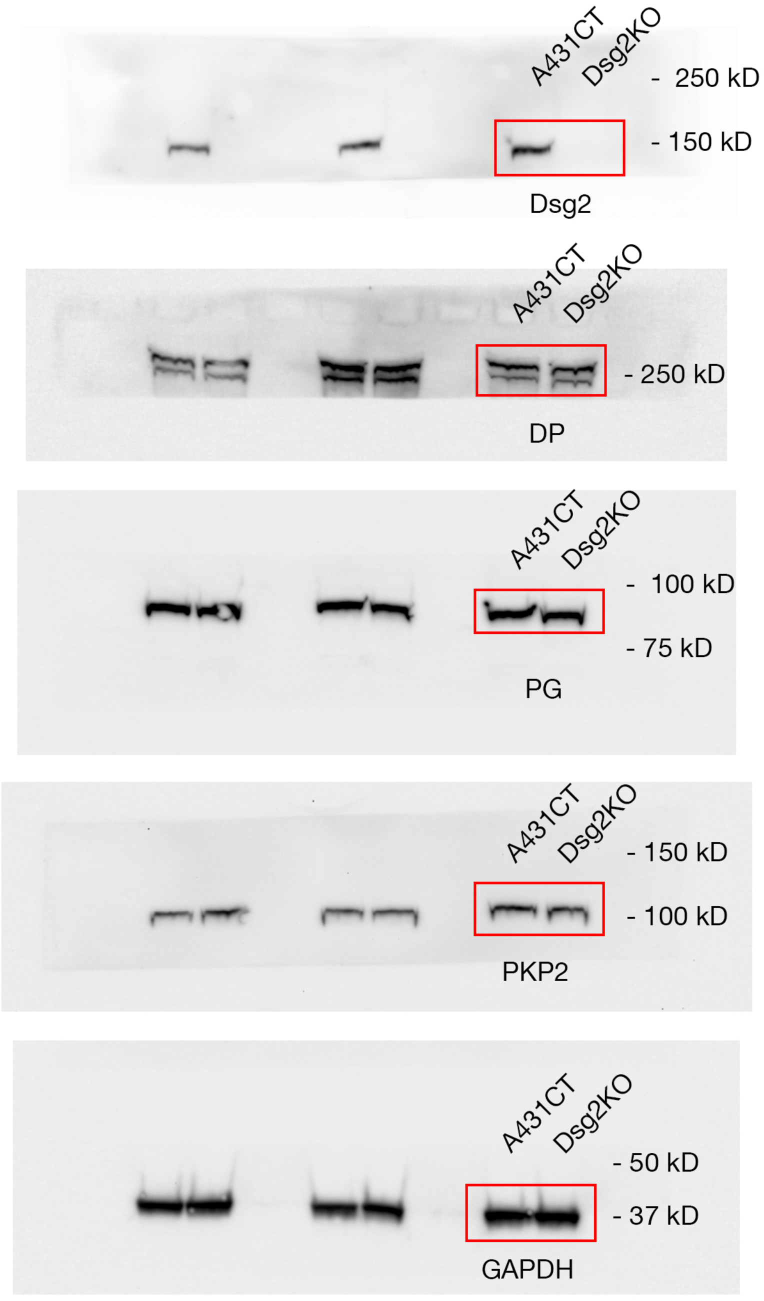

Supplementary Figure S5, Shelton WT *et. al.*

Unprocessed blot Images - Figure 3a

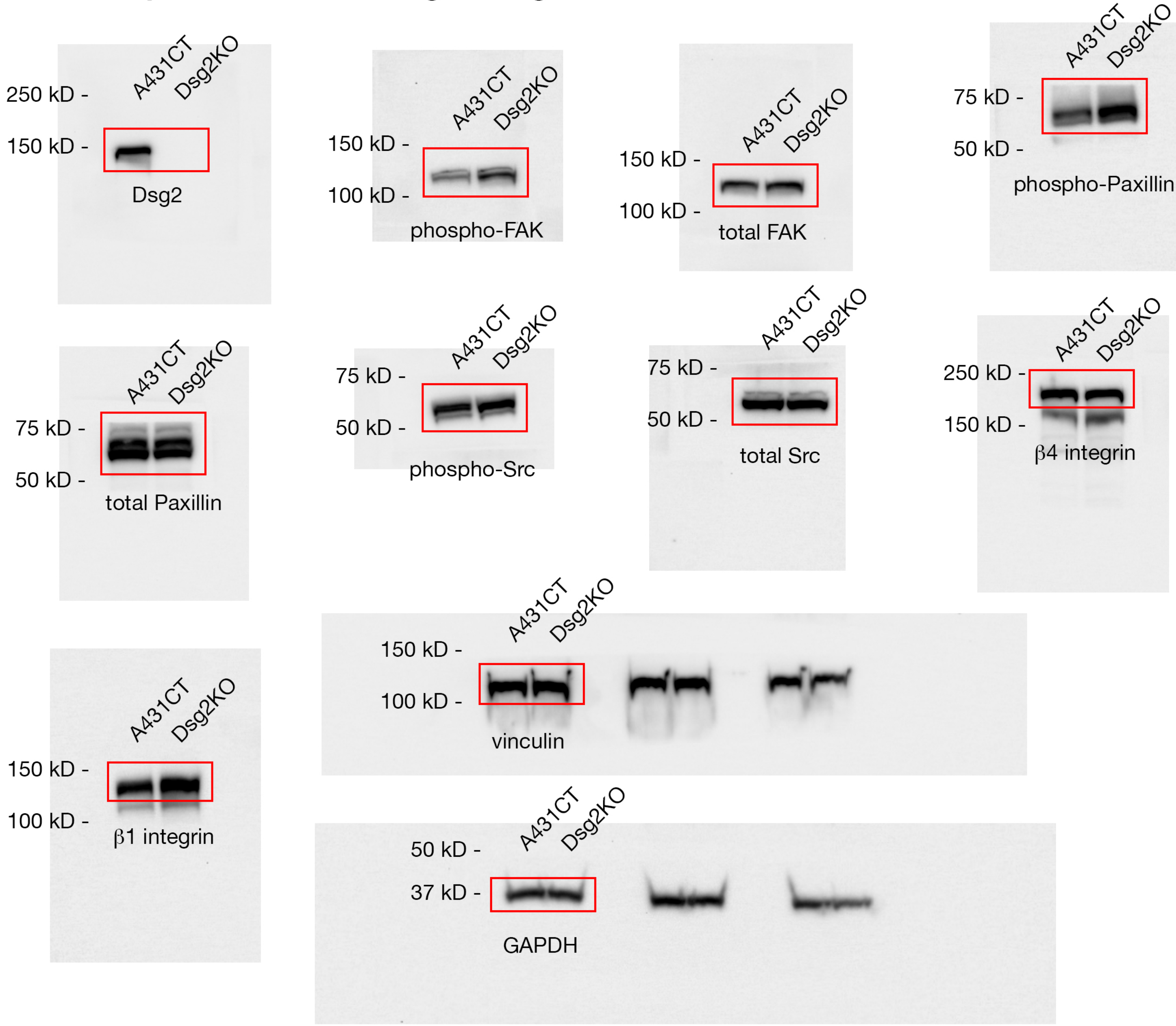

Unprocessed blot Images - Figure 3e

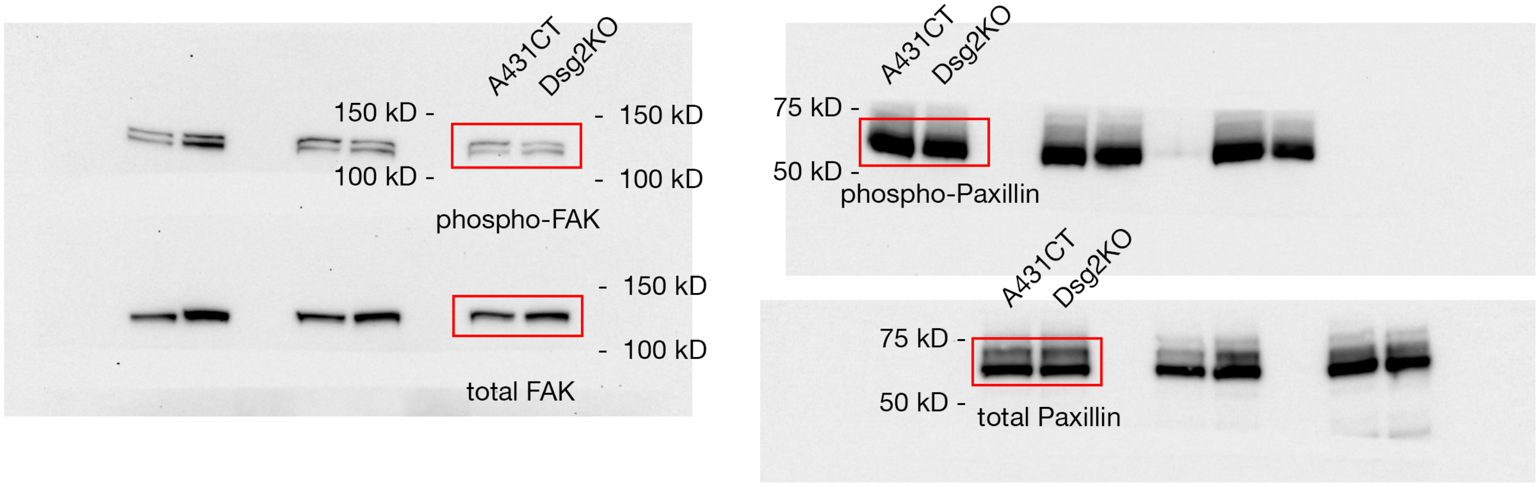

Supplementary Figure S6, Shelton WT *et. al.*

Unprocessed blot Images - Figure 4a

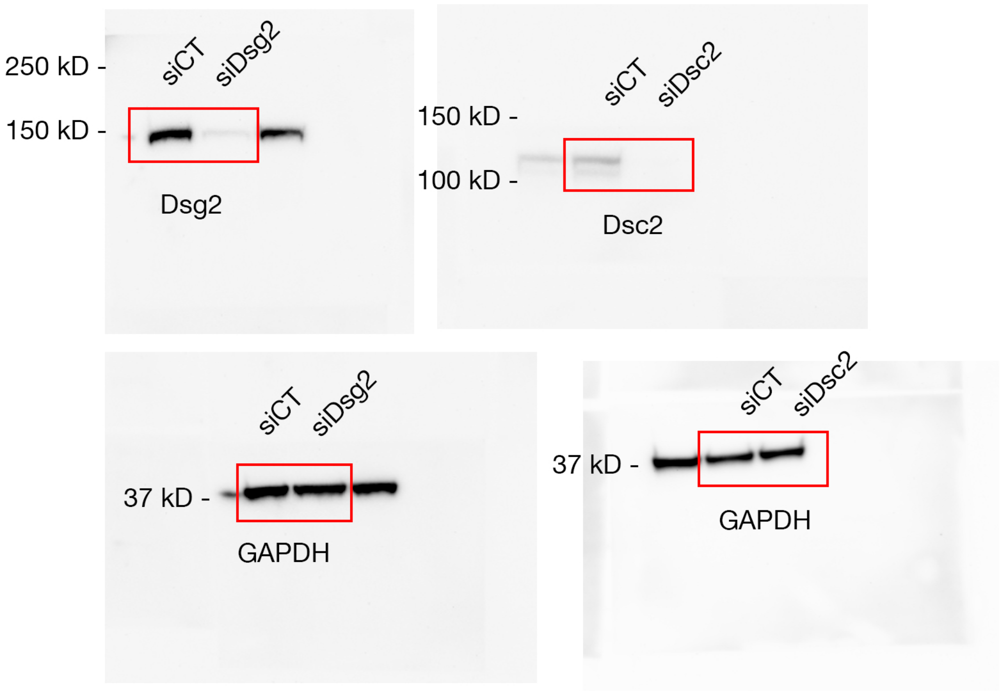

Unprocessed blot Images - Figure 4c

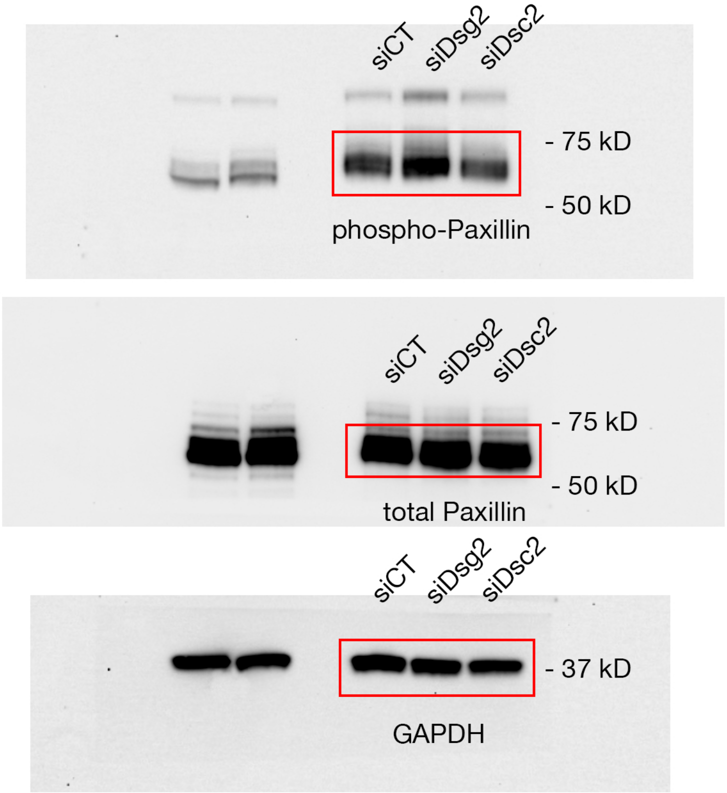

Unprocessed blot Images - Figure 4d

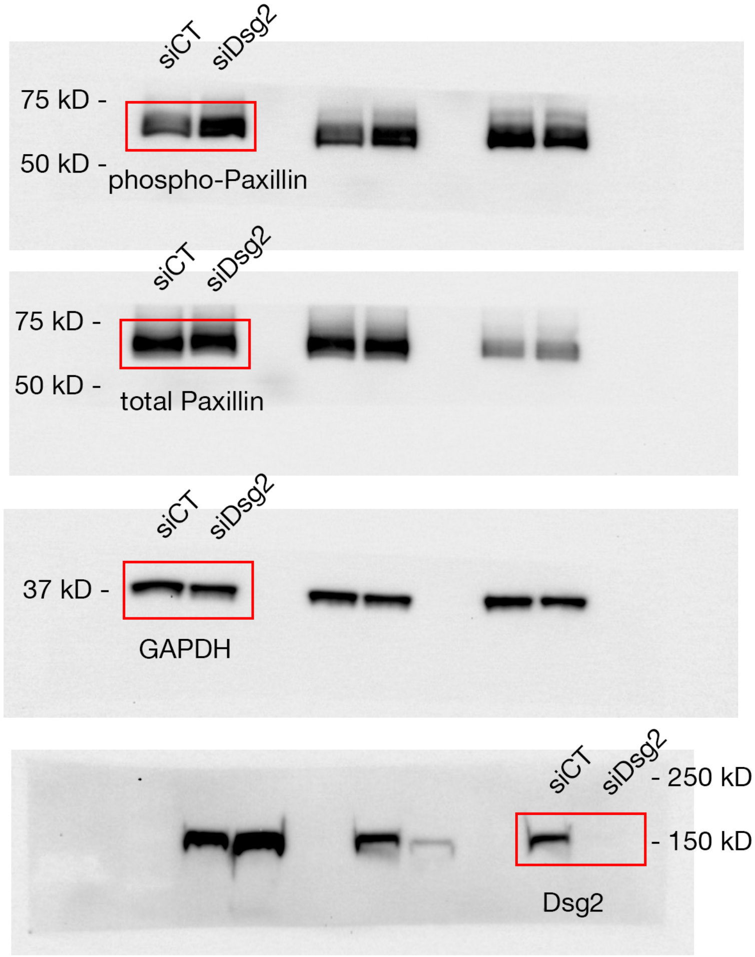

Supplementary Figure S7, Shelton WT *et. al.*

Unprocessed blot Images - Figure 5a

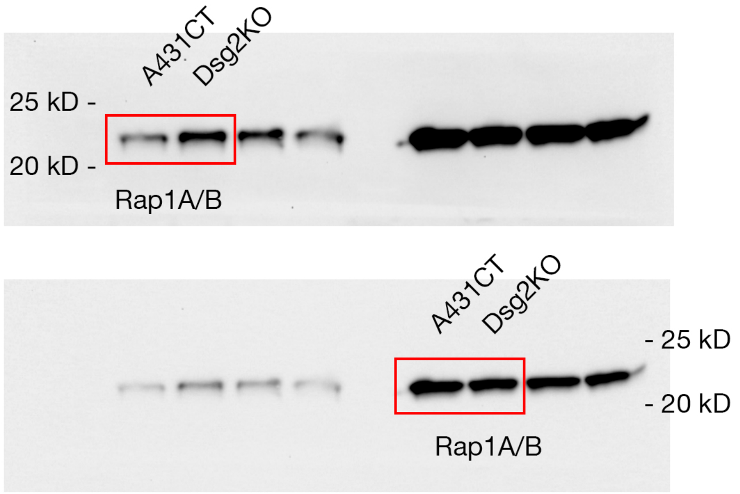

Unprocessed blot Images - Figure 5b

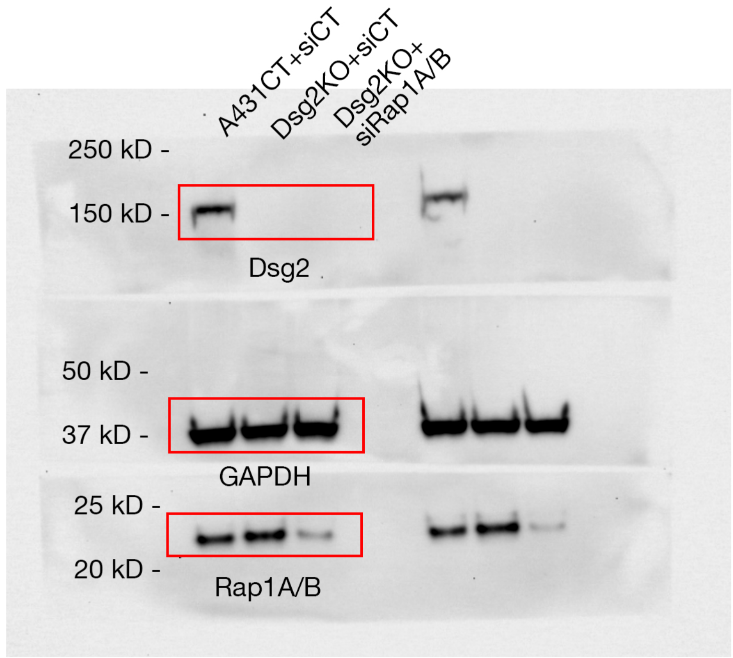

Unprocessed blot Images - Figure 5e

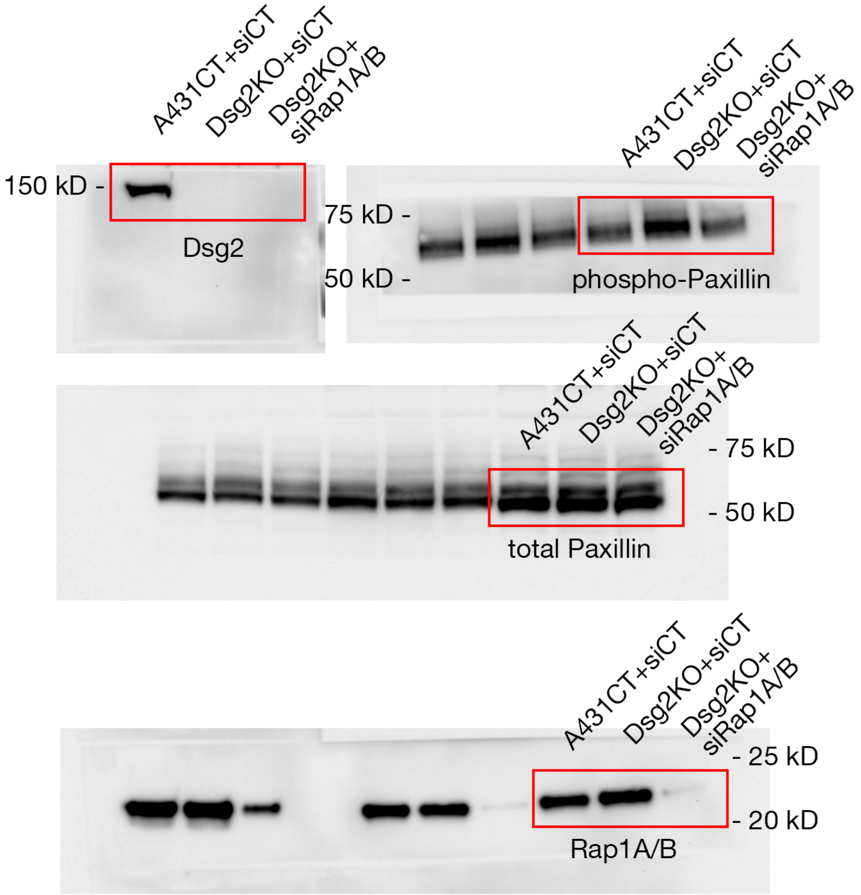

Unprocessed blot Images - Figure 6c

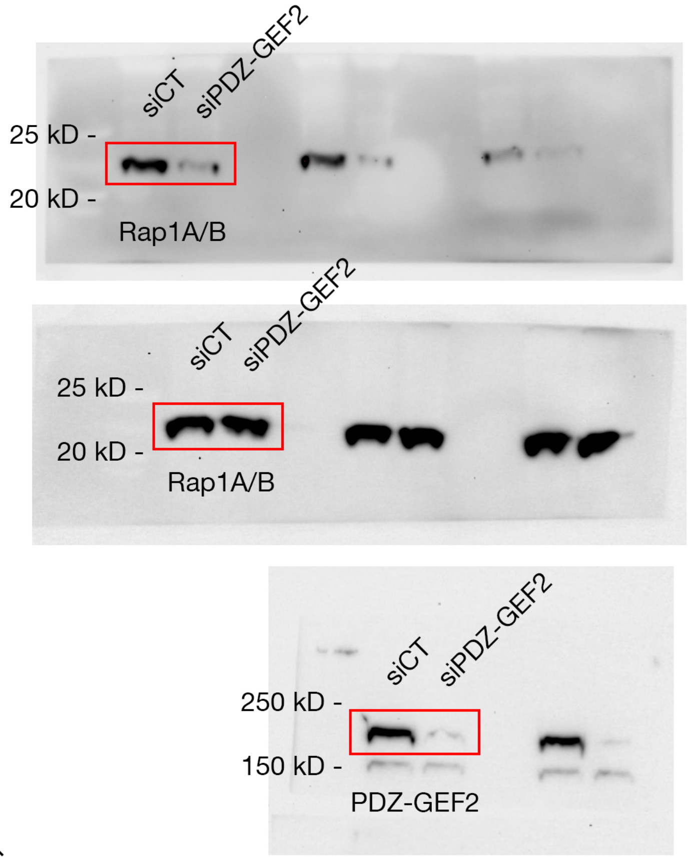

Unprocessed blot Images - Figure 6d

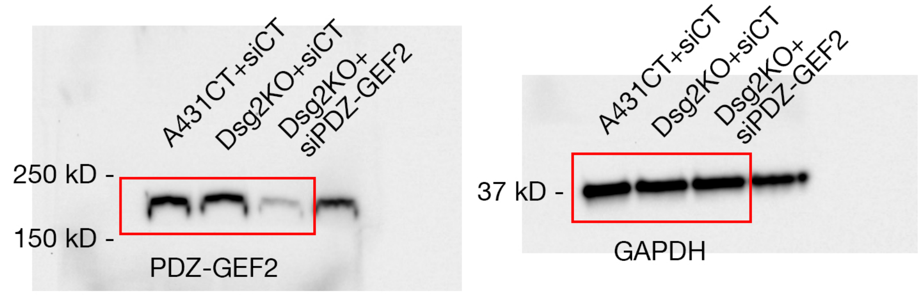

Supplementary Figure S8, Shelton WT *et. al.*

Unprocessed blot Images - Figure 7a

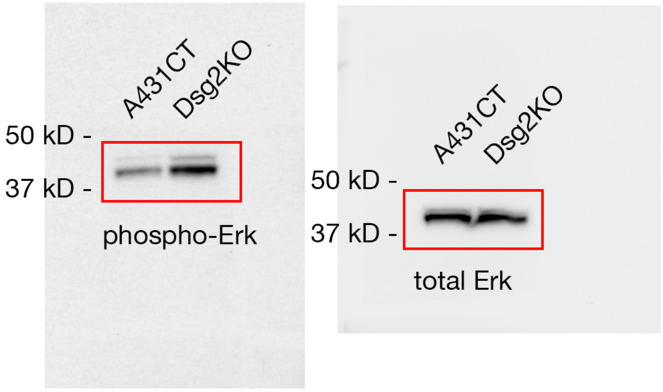

Unprocessed blot Images - Figure 7b

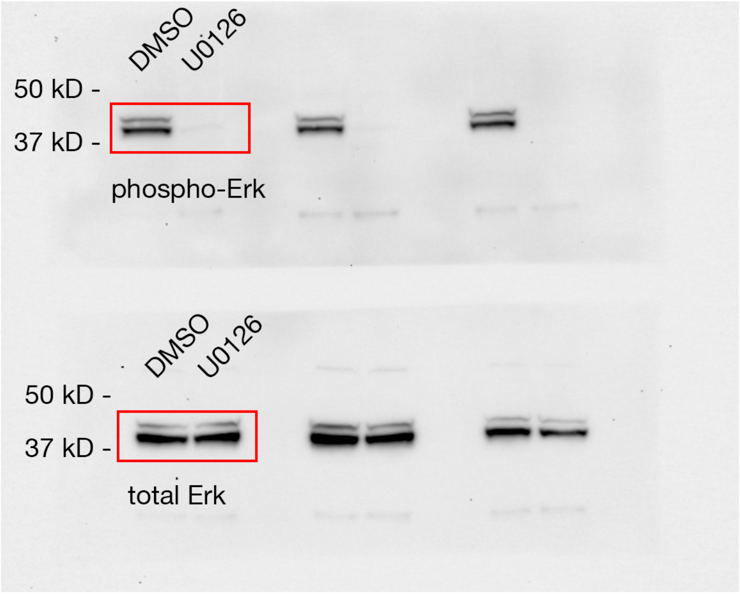

Unprocessed blot Images - Figure 8b

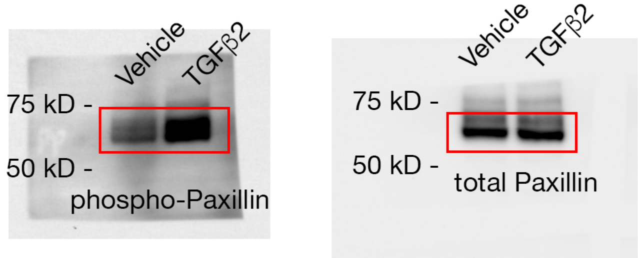

Unprocessed blot Images - Figure 8d

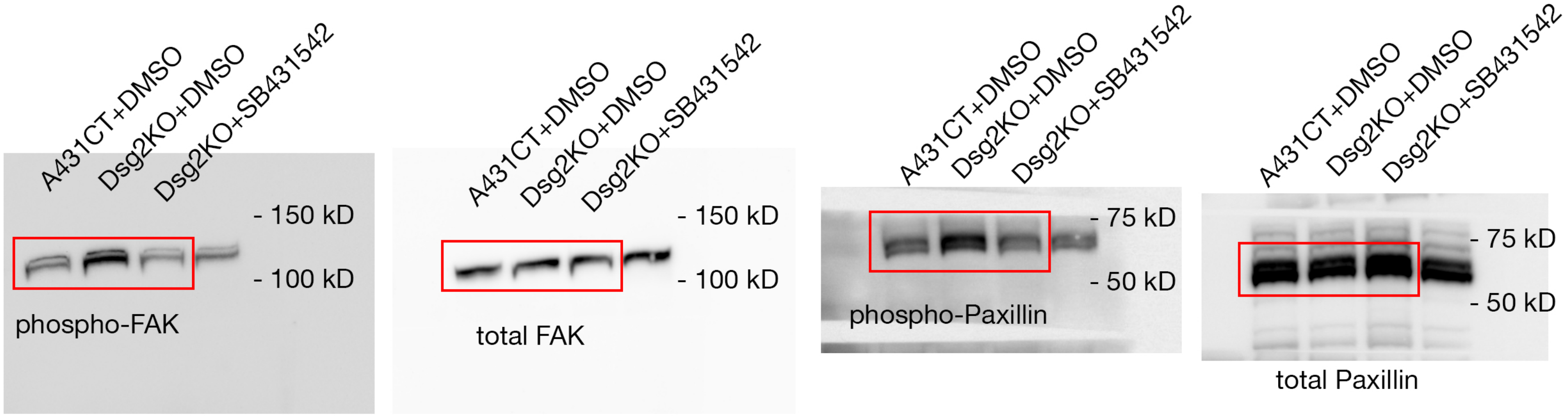

Unprocessed blot Images - Supplementary Figure S2b

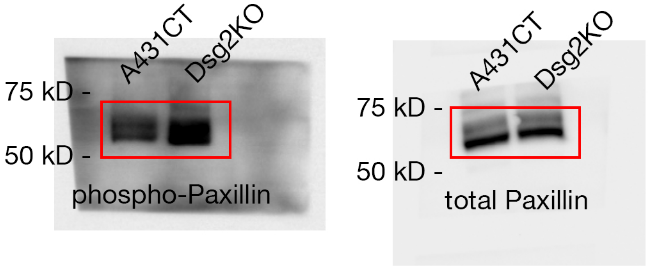

Supplement: Supplementary file 1 — Supplementary Information. [file 41598_2021_92675_MOESM1_ESM.pdf]
